# A single-cell atlas of the multicellular ecosystem of primary and metastatic hepatocellular carcinoma

**DOI:** 10.1038/s41467-022-32283-3

**Published:** 2022-08-06

**Authors:** Yiming Lu, Aiqing Yang, Cheng Quan, Yingwei Pan, Haoyun Zhang, Yuanfeng Li, Chengming Gao, Hao Lu, Xueting Wang, Pengbo Cao, Hongxia Chen, Shichun Lu, Gangqiao Zhou

**Affiliations:** 1 Department of Genetics & Integrative Omics, State Key Laboratory of Proteomics, National Center for Protein Sciences, Beijing Institute of Radiation Medicine, 100850 Beijing, China; 2 Department of Hepatobiliary Surgery, Chinese PLA General Hospital, 100853 Beijing, China; 3 Hebei University, Baoding, 071002 Hebei China; 4 Collaborative Innovation Center for Personalized Cancer Medicine, Center for Global Health, School of Public Health, Nanjing Medical University, Nanjing, 211166 Jiangsu China

**Keywords:** Cancer microenvironment, Hepatocellular carcinoma, Tumour immunology, Monocytes and macrophages, Lymphocyte differentiation

## Abstract

Hepatocellular carcinoma (HCC) represents a paradigm of the relation between tumor microenvironment (TME) and tumor development. Here, we generate a single-cell atlas of the multicellular ecosystem of HCC from four tissue sites. We show the enrichment of central memory T cells (T_CM_) in the early tertiary lymphoid structures (E-TLSs) in HCC and assess the relationships between chronic HBV/HCV infection and T cell infiltration and exhaustion. We find the *MMP9*^+^ macrophages to be terminally differentiated tumor-associated macrophages (TAMs) and PPARγ to be the pivotal transcription factor driving their differentiation. We also characterize the heterogeneous subpopulations of malignant hepatocytes and their multifaceted functions in shaping the immune microenvironment of HCC. Finally, we identify seven microenvironment-based subtypes that can predict prognosis of HCC patients. Collectively, this large-scale atlas deepens our understanding of the HCC microenvironment, which might facilitate the development of new immune therapy strategies for this malignancy.

## Abstract (web summary)

The immune cell constituents and localisation within human hepatocellular carcinoma is not fully understood. Here the authors use single cell RNA sequencing of HCC from four different tissue sites and show differences between primary and metastatic tumours, tumour associated macrophages and immune cell populations.

## Introduction

Hepatocellular carcinoma (HCC) is the most frequent primary liver cancer and is the third leading cause of cancer deaths^1^. HCC is typically resistant to chemotherapy and radiotherapy, and treatment with sorafenib or regorafenib is of limited clinical benefit^2^. Cancer immunotherapies such as immune checkpoint blockade have dramatically advanced the oncological treatment landscape over the past decades; however, the treatment options for HCC are still limited and the response rates remain low^3^. It is, therefore, paramount to characterize the baseline landscape of HCC cellular ecosystem and its key compositions associated with cancer development and immunotherapy.

Several pioneering single-cell RNA sequencing (scRNA-seq) studies have investigated the immune cells or malignant cells of primary HCCs^4–6^ and early-relapse HCCs^7^. However, these studies have not characterized a global landscape of TME combining primary and metastatic HCCs. Here, we perform a large-scale, unbiased assessment of the multicellular ecosystem of primary or metastatic HCCs from multiple tissue types. Important immune cell subtypes are identified and their relationships with tumor progression are investigated. The intratumoral heterogeneity of malignant hepatocytes and their multifaceted functions in shaping the immune microenvironment are also assessed. This large-scale transcriptomic data of single-cell resolution in HCC can be used as a resource for further exploring the basic characteristics of TME and for developing potentially effective immunotherapy strategies for this malignancy.

## Results

### scRNA-seq and cell typing of primary and metastatic HCC and paired non-tumor liver tissues

To generate a single-cell atlas of the multicellular ecosystem of HCC, we recruited 10 HCC patients with primary and/or metastatic tumors, who are representatives of different tumor-node-metastasis (TNM) stages and hepatitis virus infection status (Supplementary Fig. 1a; Supplementary Data 1). Transcriptomes of single cells were measured in four relevant tissue types of these patients, including the non-tumor liver (NTL), primary tumor (PT), portal vein tumor thrombus (PVTT) and metastatic lymph node (MLN) tissues (Supplementary Fig. 1b). Overall, we obtained the transcriptomic data of 71,915 single cells, with an average of 1979 detected genes per cell (Supplementary Data 2).

**Fig. 1:**
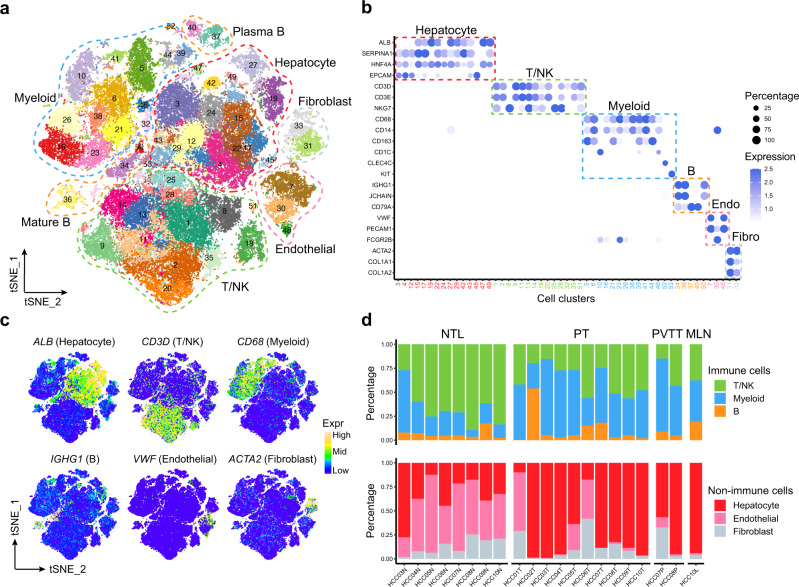
scRNA-seq profiling of multicellular ecosystem in primary and metastatic HCC and non-tumor liver tissues. **a** The T-distributed Stochastic Neighbor Embedding (tSNE) plot of 53 cell clusters from the multicellular ecosystem of 10 HCC patients. Cells from different clusters are marked by colors. Major cell types are marked by dashed lines. **b** Dotplot showing the percentage of expressed cells and average expression levels of canonical marker genes of major cell types in 53 cell clusters. Endo Endothelial, Fibro Fibroblast. **c** The tSNE plots showing the expression levels of signature genes of six major cell types, colored by gene expression. Mid, middle. **d** Stacked barplots showing the percentages of major immune (top) and non-immune (bottom) cell types in each sample. Samples are ordered based on tissue types. NTL non-tumor liver, PT primary tumor, PVTT portal vein tumor thrombus, MLN metastatic lymph node. Source data are provided as a Source Data file.

To generate a landscape of the global cellular microenvironment of primary and metastatic HCCs, we merged the scRNA-seq data across all tissues and patients using a canonical correlation analysis (CCA)-based batch correction approach. A total of 53 clusters of cells were identified using a shared-nearest neighbor (SNN)-based unsupervised clustering method (Fig. 1a and Supplementary Fig. 1c). The robustness of the clustering was tested by down-sampling and leave-one-patient-out analyses, which showed robust cluster assignments (Supplementary Fig. 1d).

We then annotated the 53 cell clusters with canonical marker genes of major cell types and found they consist of 15 hepatocyte and cholangiocyte clusters, 14 T and natural killer (NK) cell clusters, 14 myeloid cell clusters, 5 B cell clusters, 3 endothelial cell clusters and 2 fibroblast clusters (Fig. 1b, c and Supplementary Fig. 2a, b; Supplementary Data 3). We further confirmed the cell type annotation by comparing with the well-annotated cell clusters in the Human Cell Landscape (HCL) project (Supplementary Fig. 2c). We found PT showed significant depletion of T/NK cells and enrichment of myeloid cells as compared to NTL, in line with previous studies on HCC^5,6,8^. Besides, we found that PVTT and MLN tissues exhibited similar composition of major cell types with PT (Fig. 1d). To facilitate interactive exploration of the multicellular ecosystem of HCC, we created a web interface: http://omic.tech/scrna-hcc/.

### Antitumor central memory T cells are enriched in early tertiary lymphoid structures

**Fig. 2:**
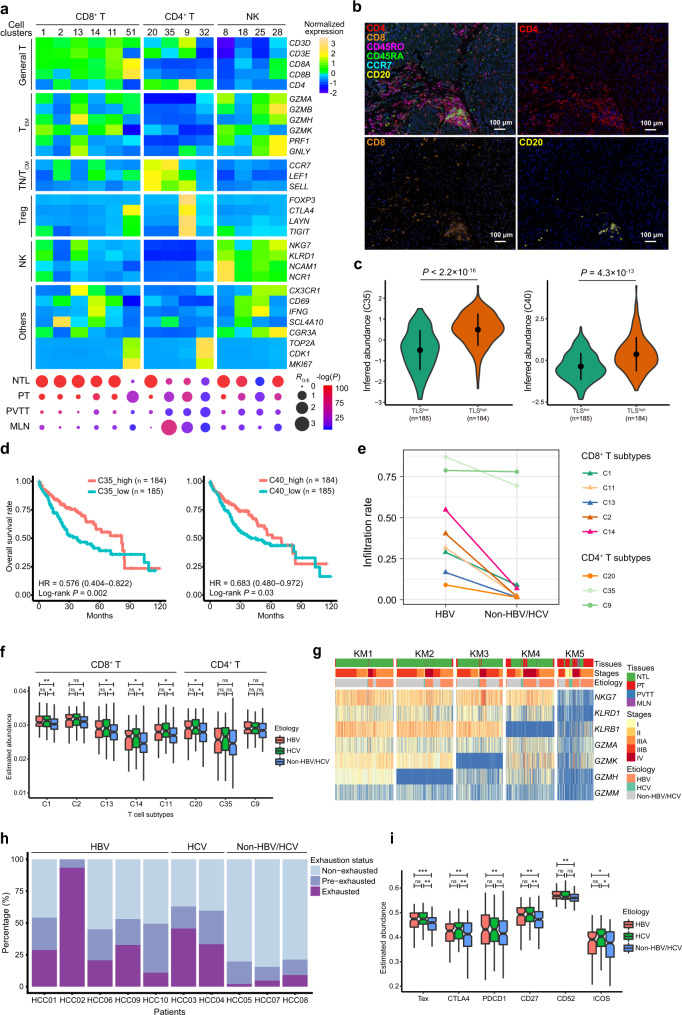
Characterization of the heterogeneous T cell populations in HCC. **a** Expression profiles of canonical marker genes of T/NK subtypes (top) and their tissue preferences (bottom). Dot size indicates the ratios of the observed *versus* expected cell numbers (*R*_O/E_); Dot color indicates the log-transformed *P* values determined by two-sided Chi-squared test. T_EM_ effector memory T, TN Naïve T, T_CM_ central memory T, Treg regulatory T, NTL non-tumor liver, PT primary tumor, PVTT portal vein tumor thrombus, MLN metastatic lymph node. **b** Multi-color IHC staining to validate the presence of CD4^+^ T_CM_, CD8^+^ T and CD20^+^ B cells aggregates in early tertiary lymphoid structures (E-TLSs) in the tumor of patient HCC03. **c** Differential abundance of CD4^+^ T_CM_ (C35) or CD20^+^ B (C40) in TLS^low^ and TLS^high^ tumors from TCGA-LIHC cohort estimated using a 9-gene TLS signature. **d** Higher abundance of CD4^+^ T_CM_ (C35) or CD20^+^ B (C40) predicts increased overall survival rates of patients in the TCGA-LIHC cohort. **e** The infiltration rates of T cell subtypes in tumors of HBV-infected (*n* = 5) and non-HBV/HCV-infected (*n* = 3) HCC patients, measured by dividing the number of T cells in tumoral tissues with the number of the whole T cell subtype. **f** The inferred abundances of T cell subtypes in HBV-infected (*n* = 104), HCV-infected (*n* = 56) and non-HBV/HCV-infected (*n* = 216) tumor samples in the TCGA-LIHC cohort. **g** The *k*-means clustering of cytotoxic T lymphocytes (CTLs) based on the expression levels of CTLs effector molecules. **h** Proportions of the exhausted, pre-exhausted and non-exhausted CTLs in HBV-, HCV- or non-HBV/HCV-infected patients. **i** The differential abundances of exhausted T cells (Tex) and differential expression levels of exhaustion markers in HBV-infected (*n* = 104), HCV-infected (*n* = 56) and non-HBV/HCV-infected (*n* = 216) tumor samples in the TCGA-LIHC cohort. In **c**, **f** and **i**, the statistical significance was determined by two-sided Student’s *t* test. ns, not significant, *P < 0.05, ^**^P < 0.01 and ^***^P < 0.001. Boxplot elements are defined in the Methods section (data visualization). Source data are provided as a Source Data file.

We identified a total of 25,591 T/NK cells that were divided into 14 clusters (Fig. 2a). CD8^+^ T cell clusters include the cytotoxic T lymphocytes (CTLs) (C1), mucosal-associated invariant T (MAIT) cells (C2), effector memory T (T_EM_) cells (C13), and tissue-resident memory T (T_RM_) cells (C14). Cells in cluster C11 are associated to an intermediate state (T_Int_) between the naïve T (TN)/central memory T (T_CM_) cells and CTLs according to the trajectory analysis (Supplementary Fig. 3a). We found most CD8^+^ T and NK subtypes are enriched in non-tumor livers and depleted in primary and metastatic tumors (Fig. 2a), distinct from the tissue preferences of T cells in breast^9^ or lung cancer^10^. Nonetheless, the infiltration levels of intratumoral CD8^+^ T and NK cells showed heterogeneity across patients, varying from 1.8% to 27.8% (Supplementary Fig. 3b).

Among CD4^+^ T cell subtypes, regulatory T cells (Tregs; C9) were enriched in PT, consistent with previous findings^4,5,11^. However, we identified another cluster of CD4^+^ T cells (C35), highly expressing key markers of TN or T_CM_ cells, were specifically enriched in PT and MLN. Using multi-color immunohistochemistry (IHC) assays, we confirmed these cells to be CCR7^+^CD45RA^−^CD45RO^+^ T_CM_ (Supplementary Fig. 3c, d). In line with this, comparison with T cell subtypes identified by other studies showed that these cells shared the highest similarity with ANXA1^+^CD4^+^ T cells, which were annotated as T_CM_ both in the non-small-cell lung cancer (NSCLC)^12^ and colorectal cancer (CRC)^13^ datasets (Supplementary Fig. 4a). Notably, we repeatedly observed that these intratumoral T_CM_ cells were organized in aggregates (Fig. 2b and Supplementary Fig. 3d), which resemble early tertiary lymphoid structures (E-TLSs). We then sought to determine whether there is coexistence of T and B cells aggregates in these intratumoral structures, which is a hallmark of E-TLS^14,15^. Indeed, the coexistence of CD8^+^ T, CD4^+^ T_CM_ and CD20^+^ B cell aggregates is detected in four out of nine patients assessed by immunohistochemistry (IHC) assays (Fig. 2b and Supplementary Fig. 3d). In accordance with this, we identified a cluster of B cells (C40), which highly expressed *MS4A1* (i.e., *CD20*) and *CD79A*, to be also specifically enriched in PT and MLN, closely resembling the tissue preference of CD4^+^ T_CM_ in C35 (Supplementary Fig. 4b). To further validate these findings, we classified the HCC tumors from two independent HCC cohorts (TCGA-LIHC and Fudan) into two groups: TLS^high^ and TLS^low^ and found that the inferred abundances of CD4^+^ T_CM_ (C35) and CD20^+^ B cells (C20) are significantly higher in TLS^high^ tumors as compared to TLS^low^ ones (Fig. 2c and Supplementary Fig. 4c, d). Clinical relevance analysis showed that higher abundances of CD4^+^ T_CM_ or CD20^+^ B cells in tumors are associated with improved patient’s survival (Fig. 2d and Supplementary Fig. 4e), supporting their antitumor activities. Taken together, these results indicated the potential activity of intratumoral E-TLSs in antitumor immunity in HCC by serving as depositories of antitumor T_CM_ and CD20^+^ B cells.

### Distinct T cell states between the HBV-related and non-HBV/HCV-related HCCs

Tissue preference analysis between the patients with different viral etiologies showed that most T cell clusters show higher infiltration levels in HBV-related tumors than in non-HBV/HCV-related tumors (Fig. 2e). Consistent with the scRNA-seq data, the inferred abundances of most CD8^+^ T subtypes are significantly higher in HBV- or HCV-related tumors than in non-HBV/HCV-related tumors in the TCGA-LIHC cohort (Fig. 2f). In addition, flow cytometry analyses in eleven independent HCC patients also confirmed this finding (Supplementary Fig. 5a, b).

To investigate the intratumoral CTL exhaustion in HBV-related HCCs, we identified the exhausted CTLs by dividing CTLs into subclusters based on their expression profiles of CTL markers. The five CTL subclusters (KM1–5) showed a continuous loss of expression of CTL markers (Fig. 2g). Subcluster KM5 showed depletion of all CTL markers and was dominated by CTLs derived from the primary and metastatic tumors (~77.1%; *P* = 6.8 × 10^–313^, Chi-squared test). The genes upregulated in KM5 included several important T cell exhaustion markers, such as *CTLA4*, *CD27* and *PDCD1* (Supplementary Fig. 5d; Supplementary Data 4). Hence, T cells in subcluster KM5 were defined as the exhausted CTLs. Besides, we identified and defined the CTLs in subcluster KM4 to be pre-exhausted CTLs due to their loss of expression of CTL markers and sharing a high portion of signature genes with KM5 (Supplementary Fig. 5e; Supplementary Data 5). We found that the frequency of exhausted and pre-exhausted CTLs in HBV-related patients is markedly higher than that of non-HBV/HCV-related patients (Fig. 2h). We confirmed this finding in the TCGA-LIHC cohort, as either the HBV-related or HCV-related tumors harbors significantly higher abundances of exhausted CTLs compared to non-HBV/HCV-related tumors and expresses consistently higher levels of exhaustion markers, while no significant differences were observed between the HBV-related and HCV-related tumors (Fig. 2i). Flow cytometry analyses also showed that the percentages of CD8^+^PD1^+^ T cells were significantly higher in HBV-related HCC tumors as compared to non-HBV/HCV-related cases (Supplementary Fig. 5a, c). Together, these findings indicate that chronic HBV/HCV infection is relevant to the infiltration and exhaustion status of CD8^+^ CTLs in HCC tumors.

### Characterization of the heterogeneity of intratumoral macrophages in HCCs

**Fig. 3:**
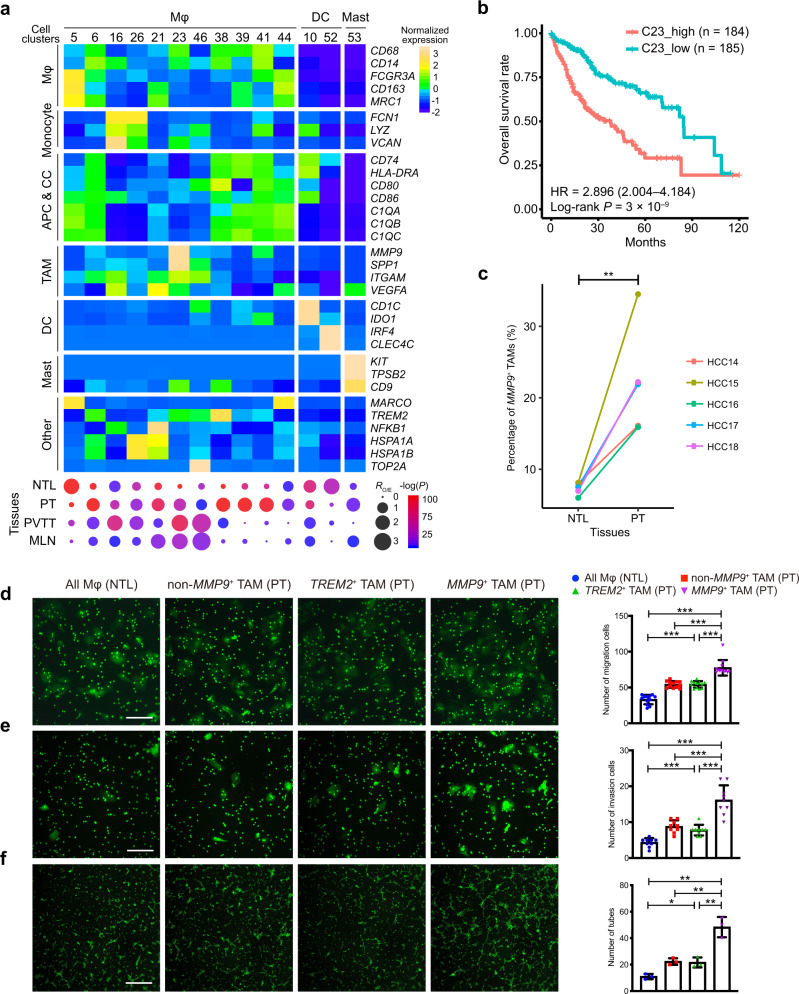
Characterization of the heterogeneity of tumor-infiltrating macrophages. **a** Heatmap (top) showing the expression profiles of canonical marker genes of myeloid cell subtypes and dotplot (bottom) showing their tissue preferences. Dot size indicates the ratios of the observed *versus* expected cell numbers (*R*_O/E_); Dot color indicates the log-transformed *P* values determined by two-sided Chi-squared test. APC antigen-presenting cell, CC complement cascade, DC dendritic cell, TAM tumor-associated macrophage, Mast mastocyte, Mφ macrophage. **b** Higher abundances of *MMP9*^+^ TAMs (C23) in tumors predict worse overall survival rates in HCC patients from the TCGA-LIHC cohort. Hazard ratio (HR) (with 95% confidence interval in brackets) was calculated using a Cox proportional hazards regression model, and the statistical significance was determined by log-rank test. **c** The proportions of *MMP9*^+^ TAMs in primary tumor (PT) tissues are significantly higher than those in non-tumor liver (NTL) tissues, which were measured by fluorescence-activated cell sorting (FACS) in five HCC patients. The statistical significance was determined by two-sided paired Student’s *t* test. ^**^P < 0.01. Huh7 cells treated with *MMP9*^+^ TAMs isolated from the PT tissues show increased abilities of **d** migration (*n* = 12 biological replicates) and **e** invasion (*n* = 9 biological replicates) compared to the control groups treated with the *TREM2*^+^ TAMs, non-*MMP9*^+^ TAMs isolated from the PT tissues and the whole macrophage populations isolated from the NTL tissues, respectively. Error bars, mean ± sd. **f** Human umbilical vein endothelial cells (HUVECs) treated with the *MMP9*^+^ TAMs show more tubes formation than the control groups treated with the *TREM2*^+^ TAMs, non-*MMP9*^+^ TAMs isolated from the PT tissues and the whole macrophage populations isolated from NTL tissues, respectively (*n* = 3 biological replicates). The scale bars represent 20 μm. Error bars, mean ± sd. In **d**–**f**, the statistical significance was determined by two-sided Student’s *t* test. *P < 0.05, ^**^P < 0.01 and ^***^P < 0.001. Source data are provided as a Source Data file.

We identified a total of 15,947 myeloid cells that were divided into 14 clusters, of which 11 were macrophage clusters (Fig. 3a). Most of the macrophage clusters were enriched in primary and/or metastatic tumors, except for a *MARCO*^+^ macrophage cluster (C5) enriched in NTL. This led to a large number of heterogeneous intratumoral macrophages (*n* = 9445), making up ~46% of the immune cells in tumors, consistent with the flow cytometry observations (Supplementary Fig. 6a, b). High inter-tumoral heterogeneity of macrophages was also observed, as four macrophage clusters (C38, C39, C41 and C44) were specifically associated to individual patients (Supplementary Data 3).

We then focused on the five macrophage clusters (C6, C16, C26, C21 and C23) enriched in tumor tissues and shared across the patients. Macrophages in C6 cluster highly express *TREM2*, an anti-inflammatory regulator specifically expressed in infiltrating macrophages recruited by inflammation^16^, suggesting that these *TREM2*^+^ macrophages in C6 may be a set of anti-inflammatory macrophages newly recruited into tumors. Cluster similarity analysis showed that *TREM2*^+^ TAMs (C6) shared a high similarity with *C1QC*^+^ TAMs and *FOLR2*^+^ TAMs in the pan-cancer^8^ and Sharma’s HCC^6^ datasets respectively. Macrophages in C16 and C26 clusters express relatively low levels of macrophage markers (*CD68*, *CD14* and *FCGR3A*) and high levels of monocyte markers (*FCN1*, *LYZ* and *VCAN*), indicating they are monocyte-derived macrophages (MoMFs). Macrophages in C21 express high level of *VEGFA*, a well-known marker of TAM^17^, and oxidative stress-responsive genes (*NFKB1*, *HSPA1A* and *HSPA1B*), suggesting these *VEGFA*^+^ macrophages are TAMs associated to oxidative stress in tumors. In addition to C21, the macrophages in C23 are characterized by high expression levels of a different set of TAM-related molecules, including *MMP9*, *SPP1*^17^ and *ITGAM* (i.e., *CD11b*)^18^, suggesting these *MMP9*^+^ macrophages are another set of TAMs different from the *VEGFA*^+^ TAMs. Similarity analysis showed that the *MPP9*^+^ TAMs (C23) shared a mild similarity with *SPP1*^+^ TAMs in Sharma’s HCC dataset (Supplementary Fig. 6c, d). We further examined the clinical relevance of these two subtypes of TAMs in two additional HCC cohorts and found that higher abundance of *MMP9*^+^ TAMs in tumors is strongly associated with worse overall survival (Fig. 3b and Supplementary Fig. 6e), as compared to a weak association of *VEGFA*^+^ TAMs (Supplementary Fig. 6f).

This promoted us to further evaluate the functions of *MMP9*^+^ TAMs in HCC progression. *MMP9*^+^ TAMs were sorted from the HCC primary tumors using flow cytometry by gating on their cell surface markers identified in the scRNA-seq data (*CD45*^+^*CD68*^+^*CD11b*^+^*MMP9*^+^; Supplementary Fig. 6a**)**. We found that the fractions of *MMP9*^+^ TAMs in PT are significantly higher than those in NTL (Fig. 3c), consistent with the scRNA-seq data. We observed that co-culturing with *MMP9*^+^ TAMs significantly promoted the migration and invasion of HCC cells and the tube formation of human umbilical vein endothelial cells (HUVECs) (Fig. 3d–f and Supplementary Fig. 7a, b), suggesting that the *MMP9*^+^ TAMs could promote HCC progression through inducing HCC cells migration, invasion, and tumor angiogenesis.

### *MMP9*^+^ macrophages are terminal TAMs that are differentiated from distinct subpopulations

**Fig. 4:**
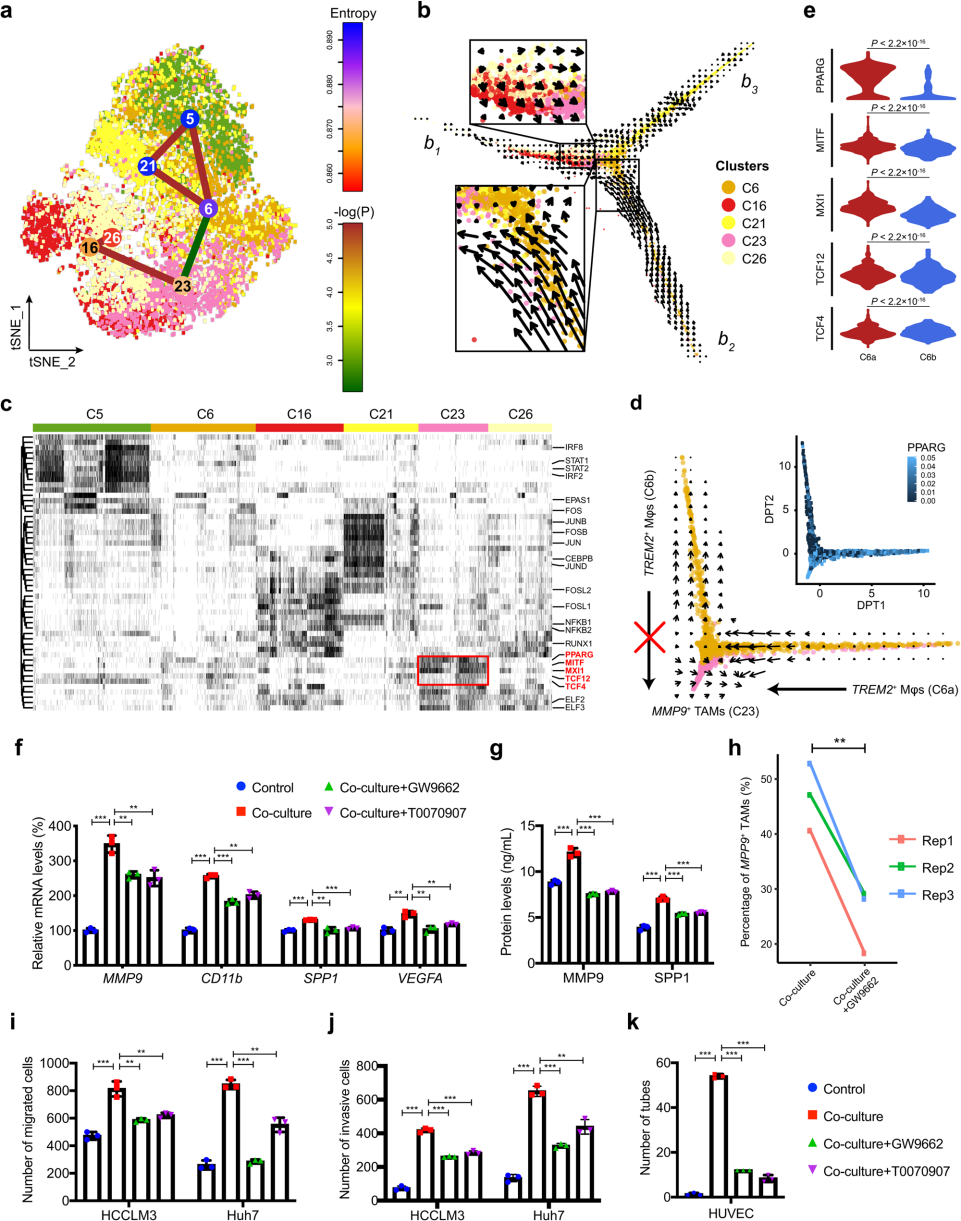
The differentiation of *MMP9*^+^ TAMs from distinct macrophage subpopulations is induced by PPARγ. **a** Macrophage lineage reconstruction by StemID2. Transcriptome entropy of each macrophage cluster is denoted by node color. Significance of links is calculated as described in the Methods section (StemID-based cellular lineage analysis) and is denoted by edge color. **b** RNA velocities are visualized on the diffusion pseudotime (DPT) projection of tumor-infiltrating macrophages. Arrows indicate the RNA velocity flow. **c** Heatmap showing the active status of transcription factors (TFs) across macrophage clusters. TFs specifically activated in *MMP9*^+^ TAMs (C23) are marked by the red box. **d** RNA velocities between the *MMP9*^+^ (C23) and *TREM2*^+^ macrophages (C6) are visualized on DPT projection. Differential activity of PPARG in two *TREM2*^+^ macrophage subpopulations (C6a and C6b) is visualized by heatmap. Mφ, macrophage; TAM, tumor-associated macrophage. **e** The differential activities of five *MMP9*^+^ TAM-related TFs between two *TREM2*^+^ macrophage subpopulations (C6a and C6b). The statistical significance was determined by unpaired two-tailed *t* test. **f** Expression levels of *MMP9*^+^ TAMs markers measured by qRT-PCR in THP-1 macrophages cultured alone (Control), co-cultured with HCCLM3 cells (Co-culture+DMSO), or co-cultured in the presence of the PPARγ inhibitors GW9662 (Co-culture+GW9662) or T0070907 (Co-culture+T0070907). **g** Protein levels of *MMP9*^+^ TAMs markers MMP9 and SPP1 measured by ELISA in the culture media of THP-1 macrophages of different groups. **h** The proportion of *MMP9*^+^ macrophages in co-cultured THP-1 macrophages without treatment of GW9662 are significantly higher than those in THP-1 macrophages treated by GW9662, measured by FACS. The statistical significance was determined by two-sided paired Student’s *t* test. Migration **i** and invasion **j** abilities of HCCLM3 and Huh7 cells treated with different sets of THP-1 macrophages. **k** Number of tubes formed by HUVECs treated with different sets of THP-1 macrophages. In **f**–**k**, data was collected from 3 biological replicates. In **e**–**g**, **i**–**k**, the statistical significances were determined by two-sided unpaired Student’s *t* test. Error bars in **f**, **g**, **i**–**k** indicate mean ± sd. *P < 0.05, ^**^P < 0.01 and ^***^P < 0.001. Source data are provided as a Source Data file.

Next, we sought to explore the differentiation trajectories among these heterogeneous macrophage subpopulations. StemID2 was used to reconstruct the cellular lineage by exploiting the tree topology and transcriptome composition of single cells^19^ (Methods). This analysis showed two fully connected meta-clusters of macrophages (Fig. 4a). The first meta-cluster includes *MARCO*^+^ (C5), *TREM2*^+^ (C6) and *VEGFA*^+^ macrophages (C21), and the second one includes two clusters of MoMFs (C16 and C26). Notably, we found *MMP9*^+^ TAMs (C23) is connected with both meta-clusters, suggesting its close relationships with both meta-clusters. To further validate these relationships, an orthogonal algorithm, RNA velocity analysis, was performed and displayed in a diffusion pseudotime (DPT) space. We found that *MMP9*^+^ TAMs at the center of DPT map serve as a hub and are connected with three branches (*b*_1_–*b*_3_; Fig. 4b). Among these branches, *b*_1_ and *b*_2_, which are composed of MoMFs and *TREM2*^+^ macrophages respectively, show clear RNA velocity flows toward the hub (i.e., *MMP9*^+^ TAMs). Thus, in light of both the lineage tree reconstruction and RNA velocity analysis, our data suggested that *MMP9*^+^ TAMs might be a set of terminally differentiated TAMs that can be accumulated through two distinct differentiation trajectories from both MoMFs and *TREM2*^+^ macrophages.

Further, we investigated the potential driver transcription factors (TFs) underlying the differentiation trajectories using SCENIC^20^. Distinct sets of TFs were activated among the heterogeneous macrophage subpopulations, and five TFs (PPARG, MITF, MXI1, TCF12 and TCF4) were specifically activated in *MMP9*^+^ TAMs (Fig. 4c). In addition, the *TREM2*^+^ macrophages contain both a subset that differentiates to *MMP9*^+^ TAMs (C6a) and a subset does not (C6b) (Fig. 4d), which nicely provided a strict case-control for looking into the key driving TFs in *MMP9*^+^ TAM differentiation. We found all the five TFs show significant increases of activities in subset C6a than in C6b (Fig. 4e). Especially, *PPARG* (encoding PPARγ) showed a threefold increase of activity. PPARγ has been shown to be required for the maturation of alternatively activated macrophages in skeletal muscle and liver^21^, and our analysis suggests it may be crucial for the terminal differentiation of *MMP9*^+^ TAMs in HCC.

Besides, we found that *MMP9*^+^ TAMs were also enriched in PVTT, so was a MoMF cluster (Fig. 3a). We then sought to explore whether the *MMP9*^+^ TAMs in PT and PVTT share the same differentiation trajectory. Interestingly, we found that the two tissue types showed different RNA velocity flows: the MoMFs show a clear flow toward *MMP9*^+^ TAMs at PT, but this trend is not obvious at PVTT (Supplementary Fig. 7c). TF analysis also showed that the activities of all the five *MMP9*^+^ TAM-related TFs are significantly reduced in PVTT than in PT (Supplementary Fig. 7d). This result suggested that *MMP9*^+^ TAMs enriched in PVTT were more likely to be recruited from PT rather than differentiated locally.

### PPARγ has a critical function in the terminal differentiation of *MMP9*^+^ TAMs in HCC

We next investigated the function of PPARγ in the differentiation of *MMP9*^+^ TAMs. We found that, both in co-cultured THP-1 macrophages or primary macrophages sorted from HCC tumors, treatment of PPARγ inhibitors, GW9662 and T0070907 significantly decreases the expression levels of marker genes of *MMP9*^+^ TAMs and the protein levels of MMP9 and SPP1 in the culture media (Fig. 4g, f and Supplementary Fig. 9a, b). In line with this, treatment of PPARγ inhibitors significantly reduced the proportion of *MMP9*^+^ TAMs in co-cultured THP-1 macrophages (Fig. 4h). Similarly, *PPARG* knockdown significantly reduced the expression levels of *MMP9*^+^ TAMs marker genes in co-cultured THP-1 macrophages and the protein levels of MMP9 and SPP1 in the culture media. Further, the effects of *PPARG* knockdown on co-cultured THP-1 macrophages were rescued by overexpression of *PPARG* (Supplementary Fig. 10a, b).

We also examined the functions of PPARγ in HCC progression through its regulation of *MMP9*^+^ TAM differentiation. We observed that the abilities of HCCLM3 and Huh7 cells migration and invasion and the tube formation of HUVECs are attenuated by PPARγ inhibitors both in THP-1 macrophages (Fig. 4i–k and Supplementary Fig. 8) and primary TAMs co-culture systems (Supplementary Fig. 9c–e). Similarly, the inhibitory effects of *PPARG* knockdown in co-cultured THP-1 macrophages on HCC cells migration and invasion and tube formation of HUVECs could be rescued by *PPARG* overexpression (Supplementary Fig. 10c–e). Collectively, these results suggested that PPARγ has a critical function in the terminal differentiation of *MMP9*^+^ TAMs in HCC, which could promote HCC progression through inducing HCC cells migration, invasion, and tumor angiogenesis.

### Intratumoral transcriptomic and genomic heterogeneity of malignant hepatocytes

**Fig. 5:**
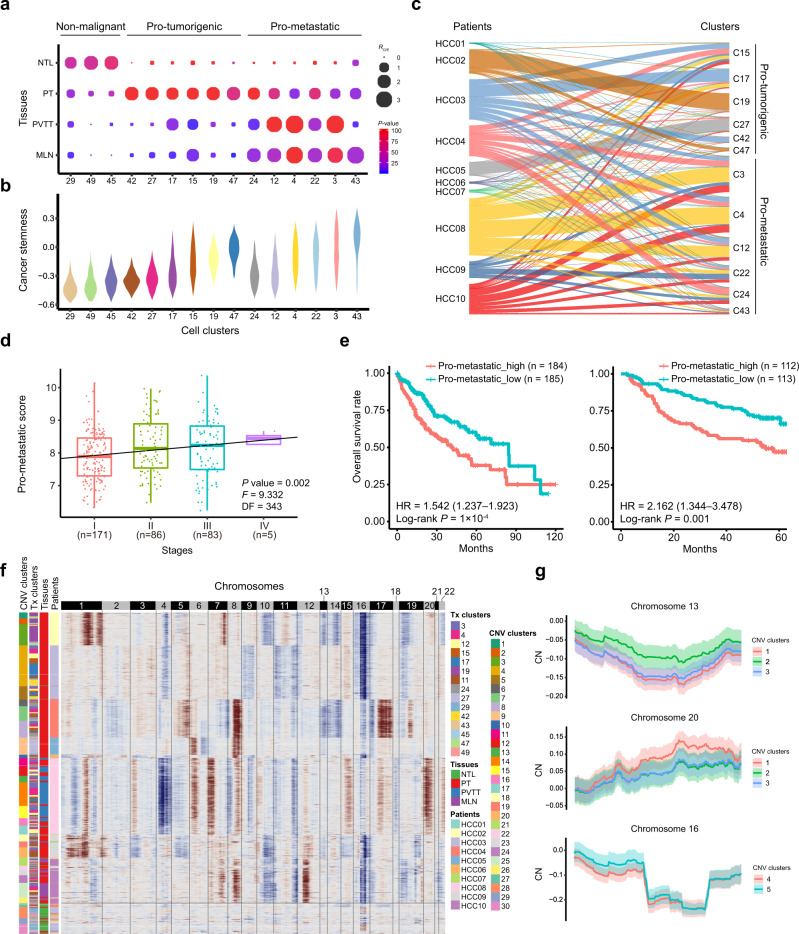
The intratumoral heterogeneity of malignant hepatocytes at the transcriptomic and genomic levels. **a** The tissue distribution preferences of three non-malignant, six pro-tumorigenic and six pro-metastatic hepatocyte clusters in NTL, PT, PVTT and MLN tissues. Dot size indicates the ratios of the observed *versus* expected cell numbers (*R*_O/E_), and dot color indicates the log-transformed *P* values determined by two-sided Chi-squared test. **b** Cancer stemness of each cell cluster was scored per cell by gene set variation analysis (GSVA) on the basis of the cancer stemness signature in hepatocyte clusters. **c** Sankey diagram showing the percentages of hepatocyte clusters among HCC patients and vice versa. **d** Significant correlation between HCC stages and pro-metastatic scores across the HCC tumors in the TCGA-LIHC cohort. A linear regression model was used for data fitting and the significance of the model was determined by two-sided F-test. DF, degree of freedom. **e** Higher pro-metastatic score of tumors predicts worse overall survival rates of HCC patients in both the TCGA-LIHC (left) and the Fudan cohorts (right). Hazard ratio (HR) with 95% confidence interval in brackets was calculated using a Cox proportional hazards regression model, and the statistical significance was determined by log-rank test. **f** Chromosomal landscape of the inferred single-cell CNV profiles in hepatocytes. By use of *k*-means clustering analyses on the basis of single-cell CNV profiles, the hepatocytes were divided into 30 CNV clusters. The number of clusters was determined based on the average silhouette width scores. Unsupervised *k*-means clustering can distinguish the malignant hepatocytes (CNV clusters 1–26) from the non-malignant ones (CNV clusters 27–30) separated by the horizontal lines. CNV, copy number variation. Tx Transcriptome. **g** Inferred copy-number (CN) profiles of subclones in patient HCC02 at chromosome 13 (top) and 20 (middle), and HCC03 at chromosome 16 (bottom). The solid curve and colored band indicate mean ± standard deviation (s.d.) of CN in each chromosomal position across single cells. Source data are provided as a Source Data file.

We identified a total of 20,406 hepatocytes that were separated into 14 clusters, enabling us to systemically explore their heterogeneity in primary and metastatic HCCs. Two clusters of hepatocytes (C29 and C49) and a cholangiocyte cluster (C45) were specifically enriched in the NTL tissues, indicating they are non-malignant cell clusters. For the remaining 12 malignant cell clusters, six clusters (C15, C17, C19, C27, C42 and C47) were specifically enriched in primary tumors, therefore designated as pro-tumorigenic hepatocyte clusters, and the other six clusters (C3, C4, C12, C22, C24 and C43) were enriched in metastatic tumors, designated as pro-metastatic hepatocyte clusters (Fig. 5a). Pathway analyses showed that these clusters are associated with the activation of distinct pathways, so did hepatocytes at different tissue sites (Supplementary Fig. 11a, b). We also found that the pro-metastatic hepatocytes showed higher levels of cancer stemness than the pro-tumorigenic hepatocytes (*P* = 1.1 × 10^–145^), while the non-malignant cells showed the lowest cancer stemness (*P* = 0.0; Fig. 5b).

Notably, we observed that the malignant hepatocytes from an individual HCC patient are often distributed across multiple clusters (Fig. 5c), rather than forming a single cluster as previously reported^22^. We attributed this difference to the larger sample size and the batch correction of our dataset. To rule out the potential artifacts in batch correction and clustering, we also performed two additional batch correction and clustering approaches and drew similar conclusions (Supplementary Figs. 12 and 13). Furthermore, we derived a list of signature genes specifically expressed by pro-metastatic hepatocytes as compared to pro-tumorigenic ones (Supplementary Data 7). By scoring HCC tumors in two independent cohorts with this signature, we found higher pro-metastatic scores are correlated with HCC stages and predict worse overall survival rates of HCC patients (Fig. 5d, e).

We also explored the heterogeneity of malignant cells at the genomic level by inferring the single-cell copy-number variation (CNV) profiles (Fig. 5f). The malignant cells from each patient could be classified into different CNV clusters, which shared globally similar CNV profiles but were significantly different at specific chromosome(s) (Fig. 5g), indicating they belong to different subclones. Despite of the high consistency on malignant cells identification, we did not observe direct correspondence between the transcriptome-based and CNV-based clusters (Supplementary Fig. 11d, e), suggesting high independence between large-scale chromosomal aberrations and transcriptomic heterogeneity of malignant cells. Together, these results highlighted the intratumoral heterogeneity of the malignant hepatocytes and their clinical implications.

### Malignant hepatocytes have multifaceted functions in shaping the microenvironment of HCC

**Fig. 6:**
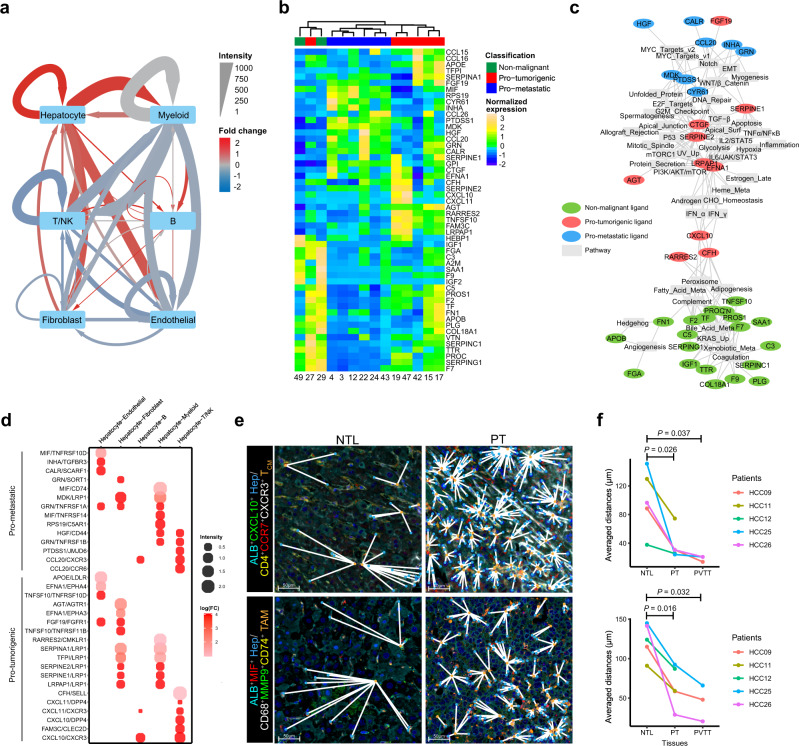
Malignant hepatocytes have multifaceted functions in shaping the HCC microenvironment. **a** A directed network showing the differential ligand-receptor (L-R) interaction intensities among the six major cell types between the primary tumor (PT) and non-tumor liver (NTL) tissues. The averaged L-R interaction intensities in the PT and NTL tissues are represented by edge width. The fold changes of L-R interaction intensities are represented by edge color, with red denoting upregulated and blue denoting down-regulated in PT as compared to NTL tissues. **b** Heatmap showing the expression levels of all hepatocyte-expressed ligands (rows) across the malignant hepatocyte clusters (columns). Ligands and malignant hepatocyte clusters are ordered based on hierarchical clustering. **c** A bipartite network linking the hepatocyte-expressed ligands (colored ellipses) to the correlated pathways (gray rectangles). Each edge indicates a significant correlation between a ligand and a pathway (*P* < 0.01, Spearman’s ρ test). Meta metabolism, EMT epithelial-mesenchymal transition, UV ultraviolet, v1 version 1, v2 version 2. **d** Dotplot showing the pro-tumorigenic and pro-metastatic L-R interactions that are significantly upregulated in PT as compared to NTL tissues. Dot size indicates the averaged L-R interaction intensity; Dot color indicates the log-transformed fold change of intensities. **e** Multi-color IHC for the co-localization analysis of interacting cell populations mediated by L-R interactions CXCL10/CXCR3 (upper) and MIF/CD74 (lower) in the non-tumoral liver (NTL) and primary tumor (PT) tissues of patient HCC26. The nearest cells between two interacting cell populations are linked by white solid lines. The experiment was repeated independently 5 times with similar results. Hep, hepatocyte; T_CM_, central memory T TAM, tumor-associated macrophage. **f** Average distances between interacting cell populations mediated by L-R pairs CXCL10/CXCR3 (upper) and MIF/CD74 (lower) in the NTL, PT and PVTT tissues of five patients (HCC09, HCC11, HCC12, HCC25, and HCC26), among whom HCC09, HCC25, and HCC26 accompanied PVTT. The statistical significances were determined by two-sided paired Student’s *t* test. *P < 0.05, ^**^P < 0.01 and ^***^P < 0.001. Source data are provided as a Source Data file.

We identified 9758, 1296 and 6687 significant ligand-receptor (L-R) interactions among the cell types that were presented in the PT, PVTT and NTL tissues, respectively (Supplementary Data 8–10). The metastatic lymph node was excluded from the analysis due to its limited number of profiled cells. We found the intensities of hepatocyte-related L-R interactions were dramatically increased in PT (Fig. 6a) and PVTT (Supplementary Fig. 14a) than in NTL tissues due to the production of a variety of ligands by malignant hepatocytes. This promoted us to investigate the ligand expression among hepatocyte clusters. Strikingly, we found that the non-malignant, pro-tumorigenic and pro-metastatic hepatocytes could be well distinguished by their ligand expression profiles, except for one pro-tumorigenic hepatocyte cluster (C27) exhibiting similar profiles with the non-malignant hepatocyte clusters (Fig. 6b).

To investigate the relationships between the ligand expression and pathway activation in hepatocytes, we linked ligands to their correlated pathways to construct a bipartite network (Fig. 6c). We found the ligands highly expressed in non-malignant, pro-tumorigenic and pro-metastatic hepatocytes are associated with activation of distinct pathways. Specifically, the ligands highly expressed in non-malignant hepatocytes are related to the physiological functions of hepatocytes, including metabolism of xenobiotic, fatty acid and bile acid, adipogenesis, and complement system; the ligands highly expressed in pro-tumorigenic hepatocytes are related to several stress-response pathways, including inflammation, interferon, p53, and apoptosis; and the ligands highly expressed in pro-metastatic hepatocytes are related to the epithelial-mesenchymal transition (EMT), MYC targets, Notch and myogenesis pathways.

We next sought to identify the tumor-enriched L-R interactions that are mediated by pro-tumorigenic and pro-metastatic hepatocytes, respectively (Fig. 6d). In line with the pathway analysis above, we found many of the L-R interactions between pro-tumorigenic hepatocytes and T cells are related to inflammation, such as CXCL10/CXCR3, CXCL10/DPP4, CXCL11/CXCR3 and CXCL11/DPP4^23^. Interestingly, the L-R interactions between the pro-metastatic hepatocytes and immune cells are enriched of interactions known to be immunosuppressive, such as CCL20/CCR6^24^, PTDSS1/JMJD6^25^, RPS19/C5AR1^26^ and MIF/CD74^27^, suggesting an important activity of pro-metastatic hepatocytes in the immunosuppressive environment of HCC. This is consistent with a pan-cancer study that observed associations between the cancer stemness and immunosuppression in a wide range of tumors^28^, given that pro-metastatic hepatocytes show significantly higher stemness than pro-tumorigenic hepatocytes (Fig. 5b). We validated two pairs of L-R interactions CXCL10/CXCR3 and MIF/CD74, which are associated to pro-tumorigenic and pro-metastatic hepatocytes respectively, using multi-color IHC staining in the paired tissues of five HCC patients (Methods). We quantified the distance between two interacting cell populations using an image-based spatial analysis method and found that the distances between CXCL10^+^ pro-tumorigenic hepatocytes and CXCR3^+^ T_CM_ cells in PT and PVTT are significantly shorter than those in NTL tissues. Similar results are also observed for MIF^+^ pro-metastatic hepatocytes and CD74^+^ MMP9^+^ TAMs (Fig. 6e, f). In concordance with the L-R analysis, the image-based assays indicated the interacting cells mediated by CXCL10/CXCR3 and MIF/CD74 more closely co-localized with each other in tumor tissues as compared to those in NTL tissues. Collectively, our results showed the different functions of the pro-tumorigenic and pro-metastatic hepatocytes in shaping the immune microenvironment of HCC.

### Bulk tissue cell type deconvolution analysis shows seven TME subtypes of HCC

**Fig. 7:**
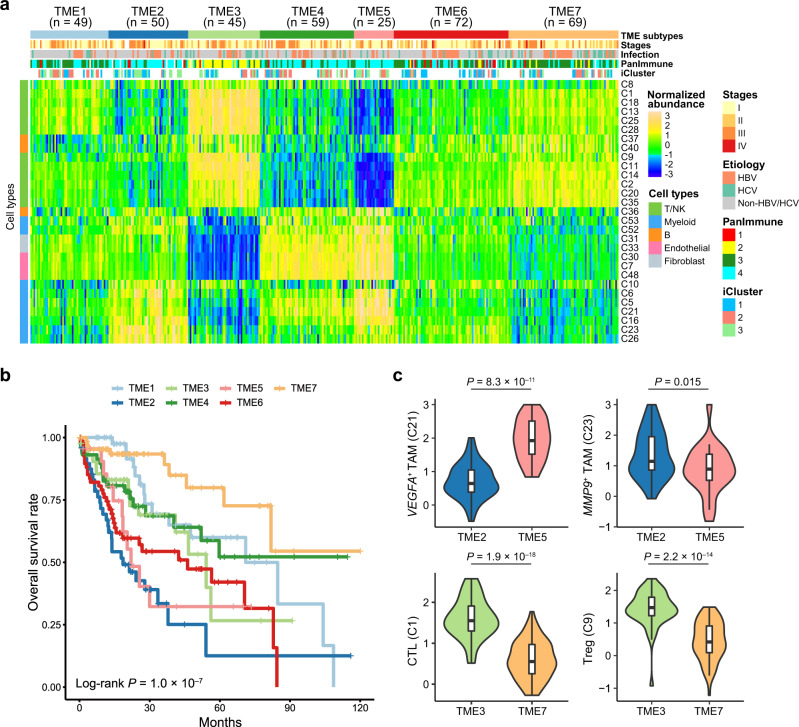
Bulk tissue cell type deconvolution analysis shows seven TME subtypes of HCC. **a** Heatmap showing seven tumor microenvironment (TME) subtypes of HCC patients (TME1–7) with distinct composition of immune and stromal cell subtypes inferred from bulk RNA-seq data of the TCGA-LIHC cohort. Rows represent the 29 patient-shared immune and stromal cell subtypes identified by the scRNA-seq data in this study. Columns represent the 369 HCC patients from the TCGA-LIHC cohort. Tumor-node-metastasis (TNM) stages, etiology, PanImmune subtypes and iCluster subtypes are annotated by colors at the top of the heatmap. **b** Kaplan–Meier survival analysis shows that the seven TME subtypes exhibit distinct overall survival outcomes. The statistical significance was determined by log-rank test. **c** The distribution of the normalized abundances of representative cell subtypes (*VEGFA*^+^ and *MMP9*^+^ TAMs, top; CTLs and Tregs, bottom) between TME subtypes TME2 (*n* = 50 patients) *versus* TME5 (*n* = 25 patients), and TME3 (*n* = 45 patients) *versus* TME7 (*n* = 69 patients), respectively. In set boxplot elements are defined in the Methods section (section on data visualization). The statistical significances were determined by two-sided Student’s *t* test. CTL cytotoxic T lymphocytes, TAM tumor-associated macrophage, Treg regulatory T cell. Source data are provided as a Source Data file.

Next, we sought to identify TME subtypes of HCC based on the abundances of immune and stromal cell subtypes inferred from the bulk RNA-seq data of the 369 HCC patients from the TCGA-LIHC cohort. The inferred abundances were normalized by dividing the total abundances of immune and stromal cells (Supplementary Fig. 15). The *k*-means clustering based on the normalized abundances shows seven distinct TME subtypes of HCC (TME1–7) (Supplementary Data 11), which are associated with significantly different clinical outcomes (*P* = 1.0 × 10^–7^, log-rank test; Fig. 7a, b).

Tumors in TME2 (*n* = 50) and TME5 (*n* = 25) subtypes exhibited a macrophage-dominated and lymphocyte-depleted microenvironment, and as expected, conferred the worst prognosis. Notably, TME2 and TME5 represented two different TAM-dominant TME subtypes. Tumors in TME2 exhibited higher proportion of *MMP9*^+^ TAMs (C23), while tumors in TME5 exhibited higher proportion of *VEGFA*^+^ TAMs (C21) (Fig. 7c). In contrast, TME7 subtype (*n* = 69) conferred the most favorable prognosis on their constituent tumors and exhibited high proportions of CTLs (C1), T_CM_ (C35) and CD20^+^ B cells (C40) and low macrophage content. Subtype TME3 (*n* = 45), which exhibited even higher proportions of CTLs but also higher proportion of suppressive Tregs than TME7 (Fig. 7c), showed a less favorable prognosis. By comparing to the previously described pan-cancer immune subtypes in PanImmune^29^, we found TME3 was specifically associated to PanImmune2 (i.e. the IFN-γ dominant subtype), consistent with the high proportions of INF-γ-producing NK (C25) and T (C14) subtypes in TME3-related tumors (Fig. 7a). We also found there is correspondence between the TME and iCluster-based molecular subtypes of HCCs^30^. TME6 subtype consisted predominantly of iCluster1 patients (*P* = 3.6 × 10^–4^), which were characterized by including a low frequency of *CDKN2A* silencing by DNA hypermethylation, a low frequency of *CTNNB1* mutations and *TERT* promoter mutations. We also found the *MMP9*^+^ TAM-enriched TME2 subtype is exclusive to iCluster2 patients (*P* = 0.0015), which exhibited a high frequency of *CDKN2A* silencing by DNA hypermethylation, a high frequency of *TERT* promoter mutations and *CTNNB1* mutations and were associated with low-grade tumors and less microvascular invasion.

## Discussion

In this study, by combining single-cell transcriptome profiling with tissue preference analysis, lineage reconstruction, TF profiling, CNV inference and cellular interaction analysis, we provided a more comprehensive landscape of the heterogeneous multicellular ecosystem in primary and metastatic HCCs.

Although much attention has been focused on T cells in the microenvironment of HCC^4^ and other types of cancer^9,10,31,32^, our data showed the enrichment of a cluster of antitumor CD4^+^ T_CM_ cells in E-TLSs in HCCs. A recent survey in 273 HCC patients showed that TLSs were presented in nearly half of the HCC tumors, among which most of them were E-TLSs, and intratumoral TLSs were significantly correlated with a lower risk of early relapse^33^. However, the mechanisms underlying the functions of TLSs in the adaptive antitumor immune response remain to be deciphered. The identification of antitumor T_CM_ enriched in E-TLSs could provide an insight of this immune-associated structure and its potential function in HCC immunotherapy. As intratumoral TLS has recently emerged as a hot topic in the era of cancer immunotherapy^34^, strategies aiming to induce/activate T_CM_ in TLSs/E-TLSs in combination with immune checkpoint inhibitors, might represent promising avenues for future cancer treatment.

Many studies have investigated the correlation between the hepatitis virus infection and T cells in peripheral systems^35,36^ or non-tumor tissues^37^. However, few studies have focused on the impact of hepatitis virus infection on intratumoral T cells. Using scRNA-seq, we found that most CD8^+^ T cell clusters are more enriched in HBV- or HCV-related HCCs as compared to non-HBV/HCV-related HCCs, and that the chronic HBV/HCV infection states are related to an increased CTLs exhaustion in HCC tumors. These findings are consistent with the high immune checkpoint blockade (ICB) efficacy of viral-associated HCC^38^, considering that higher density of CD8^+^ T cells is a classic biomarker for higher ICB response rates^39^ and higher expression of PD1 by CD8^+^ T cells was reported to be associated with increased ICB response rate^40^.

The origin and function of TAMs is one of the major concerns in the relationship between the macrophages and the development of tumors^41^. We identified *MMP9*^+^ TAMs to be a population of terminally differentiated TAMs and can be accumulated from two distinct macrophage subpopulations. One subpopulation is MoMFs, resembling CCR2^+^ inflammatory monocytes reported by a previous study^42^. Notably, besides the MoMFs, we found that the newly recruited *TREM2*^+^ macrophages are also able to differentiate to *MMP9*^+^ TAMs. Combining TF profiling and in vitro TAM differentiation assays, we confirmed that PPARγ is a driving molecule of the terminal differentiation of *MMP9*^+^ TAMs and subsequently promotes HCC progression. Nonetheless, the mechanistic linkage between PPARγ and MMP9 secretion during macrophages polarization is unclear. A series of in vivo and in vitro mechanism experiments are required to fully discover the linkage between PPARγ and MMP9 expression in macrophages.

Most scRNA-seq studies of malignant cells focused on their inter-tumoral heterogeneity^43,44^. Our results highlighted the intratumoral heterogeneity of malignant hepatocytes both at the transcriptomic and genomic levels. In line with the heterogenous nature of malignant hepatocytes, we found that the ligands highly expressed in pro-tumorigenic and pro-metastatic hepatocytes are related to inflammation and immunosuppression, respectively, suggesting the distinct functions of malignant hepatocytes in shaping the immune microenvironment of HCC. Taken together, these results suggest that the intratumoral heterogeneity of malignant cells should be considered in immunotherapy.

In summary, our comprehensive characterization of TME from different tissue sites of HCC patients showed the heterogeneous nature of immune and malignant cells in the cancer setting. Our findings indicated the differential lineage and migratory relationships of myeloid and lymphoid cells in the HCC microenvironment. Our data can be valuable resources for further investigating the biological insights of HCC, which will be helpful in developing novel therapeutic targets and/or biomarkers for current immunotherapies of this malignancy.

## Methods

### Clinical sample collection

Ten patients with primary and/or metastatic HCC were enrolled between July 2018 and December 2018 at the Chinese PLA General Hospital (Beijing, China), and their non-tumor liver (NTL), primary tumor (PT), portal vein tumor thrombus (PVTT) and metastatic lymph node (MLN) tissues were collected for single-cell RNA sequencing (scRNA-seq) and/or multi-color immunohistochemistry (IHC) assay. Besides, 16 additional HCC patients were enrolled between September 2019 and April 2022 from the same hospital, and their NTL, PT, PVTT and MLN tissues were used for flow cytometry analysis and/or multi-color IHC assay. The diagnosis of HCC and the inclusion and exclusion criteria for the patients were described in detail previously^45,46^. Briefly, all the HCC patients were newly diagnosed, pathologically confirmed, and proved not to have other types of cancer. The diagnosis of HCC was made by either positive histologic findings or an elevated serum α-fetoprotein (AFP) level (≥400 ng/mL) combined with at least one positive image on angiography, sonography, and/or high-resolution contrast computed tomography. Among the 26 HCC patients, 16 were chronic HBV carriers, who were positive for both hepatitis B surface antigen (HBsAg) and antibody immunoglobulin G to hepatitis B core antigen (HBcAb) for at least 6 months, and 2 were chronic HCV carriers, who were positive for hepatitis C antibody (HCV-Ab). Additionally, all the subjects included in this study were negative for antibodies to hepatitis D virus or human immunodeficiency virus; and had no other types of liver disease, including autoimmune hepatitis, toxic hepatitis, and primary biliary cirrhosis or Budd-Chiari syndrome. None of the patients was treated with chemotherapy, radiotherapy or any other anti-tumor medicines prior to tumor resection. This study was approved by the Research and Ethical Committee of Chinese PLA General Hospital (Beijing, China) and Beijing Institute of Radiation Medicine (Beijing, China). The informed consent was obtained from each patient or his/her guardian. The detailed demographic and clinical characteristics of these HCC patients were summarized in Supplementary Fig. 1a and Supplementary Data 1.

### Tissue dissociation and preparation of single-cell suspensions

Following surgical resection, the fresh tissues of PT, NTL, PVTT and MLN were immediately transferred to pre-cooled MACS Tissue Storage Solution (Miltenyi Biotech, Germany) and were shipped at 4 °C. For each sample, ~1 g tissue was used for dissociation, and the remaining tissue, if any, was fixed in formalin for 48 h and embedded in paraffin for subsequent immunohistochemistry analysis. For dissociation, the tissue was minced using the surgical scalpels and further disintegrated using the Liver Dissociation Kit (Miltenyi Biotech, Germany) and the gentleMACS Dissociator (Miltenyi Biotech, Germany) according to manufacturer’s instructions. The resulting single-cell suspension was filtered sequentially through sterile 70-μm and 40-μm cell strainers. The cell suspension was stained for viability with 25 mM cisplatin (Enzo Life Sciences, USA) in a 1-min-pulse before quenching with 10% FBS. The single-cell suspensions were then used for subsequent droplet-based scRNA-seq or flow cytometry analysis.

### Single-cell RNA sequencing

The single-cell suspensions were converted to barcoded scRNA-seq libraries by using the Chromium Single Cell 3’ Library, Gel Bead & Multiplex Kit and Chip Kit (10× Genomics, USA), aiming for an estimated 5000 cells per library and following the manufacturer’s instructions. Samples were processed using kits pertaining to V2 barcoding chemistry of 10× Genomics. Single samples are always processed in a single well of a PCR plate, allowing all cells from a sample to be treated with the same master mix and in the same reaction vessel. For each patient, all the samples (NTL, PT, PVTT and MLN tissues) were processed in parallel in the same thermal cycler. The generated scRNA-seq libraries were sequenced on a NovaSeq sequencer (Illumina, USA).

### Multi-color immunohistochemistry (IHC) assays

Paired tumor and non-tumor liver tissues of eleven HCC patients collected from the Chinese PLA General Hospital (Beijing, China) were used for this assay. Among the eleven HCC patients, six patients (HCC03, HCC04, HCC05, HCC06, HCC08, and HCC09) have been performed scRNA-seq on their tissue samples, and the other five (HCC11, HCC12, HCC13, HCC25, and HCC26) were additionally recruited patients (Supplementary Data 1). The specimens were collected within 30 min after the tumor resection and fixed in formalin for 48 h. Dehydration and embedding in paraffin was performed following routine methods. These paraffin blocks were cut into 5 mm slides and adhered on the slides glass. Then, the paraffin sections were placed in the 70 °C paraffin oven for 1 h before deparaffinized in xylene and then rehydrated in 100%, 90 and 70% alcohol successively. Antigen was retrieved by critic acid buffer (pH 6.0) in the 95 °C water bath for 20 min. Endogenous peroxidase was inactivated by incubation in 3% H_2_O_2_ for 15 min. Following a preincubation with 10% normal goat serum to block non-specific sites for 30 min, the sections were incubated with primary antibodies in a humidified chamber at 4 °C overnight. The antibodies used for identifying the T_CM_ were: anti-CD45RA (1:100; clone# 4KB4, ZSBIO, China), anti-CD45RO (1:100; clone# UCH-L1, ZSBIO, China), anti-CD4 (1:400; clone# EPR6855, Abcam, USA), anti-CD8 (1:500; clone# 144B, Abcam, USA), anti-CCR7 (1:500; polyclonal, Abcam, USA) and anti-CD20 (1:500; clone# L26, Abcam, USA). Multi-color IHC staining was also used to validate the cellular interactions mediated by ligand-receptor pairs. For the validation of CXCL10/CXCR3 interaction, the pro-tumorigenic hepatocytes are marked by global hepatocytes marker anti-ALB (1:1000; polyclonal, Proteintech, USA) and the expressed ligand anti-CXCL10 (1:500; polyclonal, Proteintech, USA), and the interacting T_CM_ cells are marked by T_CM_ markers anti-CD4 (1:500; clone# EPR6855, Abcam, USA), anti-CCR7 (1:200; polyclonal, Proteintech, USA) and the corresponding receptor anti-CXCR3 (1:500; clone# 1B2D6, Proteintech, USA); for the validation of MIF/CD74, the pro-metastatic hepatocytes are marked by anti-ALB (1:1000; polyclonal, Proteintech, USA) and the ligand anti-MIF (1:100, clone# 2A10-4D3, Abcam, USA), and the interacting MMP9^+^ TAMs are marked by their markers anti-CD68 (1:500; clone# KP1, ZSGB-BIO, China) and anti-MMP9 (1:100; polyclonal, Proteintech, USA), as well as the corresponding receptor anti-CD74 (1:50; clone# LN2, Abcam, USA). The antigenic binding sites were visualized using the Opal 7-Color Manual IHC Kit (PerkinElmer, USA) according to the protocol of the manufacturer. Multi-color IHC data were collected by Mantra Quantitative Pathology Workstation (PerkinElmer, USA) and analyzed by InForm 2.2.1 (PerkinElmer, USA). Cellular distances were measured using the ‘nearest neighbor analysis’ model in the HALO pathology software (Indica Labs, USA).

### Flow cytometry analysis of macrophages and cell sorting

To validate the distinct distributions of *MMP9*^+^ tumor-associated macrophages (TAMs) between the HCC tissues and NTL tissues, we conducted flow cytometry analyses on paired PT and NTL tissues of five HCC patients (Supplementary Data 1). The following antibodies were used in the flow cytometry analysis: PerCp-Cy5.5-conjugated 7-AAD (1:100; Thermo Fisher, USA), APC-H7-conjugated CD45 (1:100; clone# 2D1, BD Biosciences, USA), BV421-conjugated CD68 (1:100; clone# Y1/82A, BD Biosciences, USA), FITC-conjugated CD11b (1:100; clone# ICRF44, BD Biosciences, USA), PE-conjugated MMP9 (1:100; clone# D6O3H, CST, USA) and APC-conjugated TREM2 (1:100; clone# 237920, R&D Systems, USA). The single-cell suspensions were stained with antibodies in 2% FBS for 20 min at 4 °C, and were analyzed on an Aria II flow cytometer (BD Biosciences, USA). The expression levels of CD45, CD68, CD11b, MMP9 and TREM2 were gated by their negative controls of unstained cells and positive controls of cells stained by each antibody. For sorting the *MMP9*^+^ TAMs from the PT and NTL tissues, samples were gated for CD45^+^CD68^+^CD11b^+^MMP9^+^. For sorting the *TREM2*^+^ TAMs from the PT and NTL tissues, samples were gated for CD45^+^CD68^+^TREM2^+^. Besides, the other macrophages except for *MMP9*^+^ TAMs (referred to as non-*MMP9*^+^ TAMs) in the tumor tissues and the whole macrophage populations in the NTL tissues were sorted as negative controls by gating for CD45^+^CD68^+^CD11b^–^/CD45^+^CD68^+^MMP9^–^ and CD45^+^CD68^+^ respectively. Cells were analyzed using the BD FACSDIVA software (BD Biosciences, USA) and FlowJo software (FlowJo, USA).

### Flow cytometry analysis of T cells and sorting

To validate the distributions of CD8^+^ T cells and PD1^+^ exhausted CD8^+^ T cells between the HBV-related and non-HBV/HCV-related HCC tumors, we conducted flow cytometry analyses on seven HBV-related and four non-HBV/HCV-related HCC tumors (Supplementary Data 1). The following antibodies were used in the flow cytometry analysis: PerCp-Cy5.5-conjugated 7-AAD (1:100; Thermo Fisher, USA) APC-conjugated CD3 (1:100; clone# UCHT1, BD Biosciences, USA), FITC-conjugated CD8 (1:100; clone# RPA-T8, BD Biosciences, USA) and BV421-conjugated PD1 (1:100; clone# EH12.1, BD Biosciences, USA). The single-cell suspensions were stained with antibodies in 2% FBS for 20 min at 4 °C, and were analyzed on an Aria II flow cytometer (BD Biosciences, USA). The expression levels of CD3, CD8 and PD1 were gated by their negative controls of unstained cells and positive controls of cells stained by each antibody. Cells were analyzed using the BD FACSDIVA software (BD Biosciences, USA) and FlowJo software (FlowJo, USA).

### Cell culture preparation

To investigate the function of PPARγ in *MMP9*^+^ TAMs differentiation, both cell line and primary macrophages sorted from HCC tumors were used. For the cell line culture, human THP-1 monocytes were first induced to differentiate to THP-1 macrophages. Specifically, THP-1 monocytes were maintained in RPMI 1640 (HyClone, USA) supplemented with 10% FBS and 5 mM β-mercatoethanol and were incubated at 37 °C in a 5% CO_2_ atmosphere. To induce the THP-1 monocytes to differentiate to THP-1 macrophages, approximately 2 × 10^5^ THP-1 cells were seeded in 12-well plates and treated with 100 nM phorbol-12-myristate 13-acetate (PMA) for 24 h. The PMA-containing medium was removed after 24 h and the cells were washed three times in PBS to remove the PMA. THP-1 macrophages were then induced to differentiate to TAM-like cells by co-culture with HCCLM3 or Huh7 cells at a 12-mm transwell with 0.4 μm pore polyester membrane insert (Corning, USA) (Supplementary Fig. 7e). Specifically, approximately 2 × 10^5^ THP-1 macrophages were co-cultured with HCCLM3 or Huh7 cells that had been left to attach to the inserts for 12 h before the co-culture. These cells were co-cultured in medium containing equal volumes (1:1) of RPMI 1640 medium supplemented with 10% FBS and HCC-CM for 48 h. We found that the THP-1 macrophages co-cultured with HCC cells express significantly higher levels of *PPARG* and marker genes of *MMP9*^+^ TAMs (*MMP9*, *SPP1* and *CD11b*) as compared to non-co-cultured cells (Fig. 4f and Supplementary Fig. 7f). Accordingly, higher levels of MMP9 and SPP1 proteins were detected in the co-culture medium (Fig. 4g). Bulk RNA-seq analysis showed that the co-cultured THP-1 macrophages share the highest expression correlation with *MMP9*^+^ TAMs (C23) among all the macrophage clusters (Supplementary Fig. 7g). These results indicated that the THP-1 macrophages co-cultured with HCC cells share many properties with *MMP9*^+^ TAMs. For the primary macrophages, we sorted *MMP9*^+^ TAMs from HCC tumors in the gate of CD45^+^CD68^+^CD11b^+^MMP9^+^ for further differentiation and functional experiments.

### Knockdown and overexpression of *PPARG*

Lentiviral short harpin RNA (shRNA) constructs (pLKO.1) coding oligonucleotide sequence 5′-GTTTGAGTTTGCTGTGAAG-3′ against human *PPARG* transcript (nucleotides 1095–1113) (sh*PPARG*), and a scramble sequence 5′-GAGTGAGTAATTCATCCTG-3′ (shControl) were obtained from Inovogen Tech (Beijing, China). For rescue assays, three synonymous mutations on PPARG coding sequence were introduced in the shRNA binding site (new sequence: 5′-GTTCGAGTTCGCTGTTAAG-3′) to generate a *PPARG* construct resistant to sh*PPARG* (sh*PPARG* + *PPARG*_Δ_). Synonymous mutations were generated using the Site-Directed Mutagenesis Kit (FMTG-25, SBS Genetech, China). To generate the stable knockdown or rescue cell lines, THP-1 cells were transfected with the indicated lentiviruses, and the stable clones were selected with 2 μg/mL puromycin or 500 μg/mL G418 (Sigma, USA). Quantitative reverse transcription real-time polymerase chain reaction (qRT-PCR) assays was performed to determine the knockdown or overexpression efficiency, respectively.

### HCC cell lines and human umbilical vein endothelial cells (HUVECs) culture

The human HCC cell lines HCCLM3 and Huh7 and the human umbilical vein endothelial cells (HUVECs) were obtained from the China Center for Type Culture Collection (CCTCC; Wuhan city, China). HCCLM3 and Huh7 were maintained in high-glucose Dulbecco’s modified Eagle’s medium (DMEM; HyClone, USA) supplemented with 10% FBS (HyClone, USA), 100 U/mL penicillin, and 100 μg/mL streptomycin. The HCC-conditioned media (HCC-CM) were collected, centrifuged at 2000 × *g* at 4 °C for 10 min to remove the cell debris and stored at –80 °C. HUVECs were cultured in endothelial cell medium (ECM, ScienCell, USA) containing 5% FBS and supplemented with 100× endothelial cell growth supplement (ECGS, ScienCell, USA).

### PPARγ interference

For experiments involving in PPARγ inhibitors, the THP-1 macrophages were treated with DMSO, PPARγ inhibitors GW9662 (20 μM) or T0070907 (20 μM) for 12 h before the co-culture. For experiments involving *PPARG* knockdown and overexpression, the THP-1 macrophages transfected with sh*PPARG*, sh*PPARG* + *PPARG*_Δ_ or shControl were used for the co-culture. Then, the induced TAM-like cells were harvested to perform qRT-PCR and to co-culture with the HCC cells or HUVECs to test the abilities of cells migration, invasion and tube formation, and the co-cultured medium was collected for ELISA assay.

### qRT-PCR assays

To determine the expression levels of *PPARG*, *MMP9*, *SPP1*, *CD11b* and *VEGFA* in macrophages, total RNAs were isolated by Trizol (Invitrogen, USA) reagent and were converted to cDNAs using the superscript III First Strand Synthesis System (Invitrogen, USA). qRT-PCR assays were then performed using the Bio-Rad IQ5 System (Bio-Rad, USA). PCR reactions were performed in 20 μL reactions using the SYBR Green PCR master mix (Bio-Rad, USA) and 0.2 μM specific primers. The relative expression levels of mRNAs were calculated using the comparative C_T_ method normalized to *GAPDH*. Primers used for qRT-PCR are shown in Supplementary Data 6.

### Quantification of MMP9 and SPP1 protein levels

MMP9 and SPP1 protein levels in culture supernatants were measured using the ELISA kits purchased from R&D Systems (Cat# DMP900 and Cat# DOST00, respectively) according to the manufacturer’s instructions.

### Cells migration and invasion assays

The 24-well chemotactic camber with a polycarbonate filter of 8-μm pore size (Corning, USA) was used for cells migration assays, and the 24-well BioCoat matrigel invasion chamber (BD Biosciences, USA) was used for cells invasion assays. In brief, HCCLM3 or Huh7 cells were cultured in serum-free medium for 12 h and then stained with CellTracker Green (5-chloromethylfluorescein diacetate [CMFDA]; Invitrogen) for 30 min. For both of the cell migration and invasion assays, approximately 5 × 10^4^ stained HCCLM3 or Huh7 cells together with 5 × 10^4^ TAM/TAM-like cells in 500 μL serum-free medium were placed in the upper chamber of each well, whereas the lower chamber was loaded with 500 μL RPMI 1640 medium with 10% FBS. For assessing the effects of TAMs from clinical samples, TAMs sorted from the single-cell suspensions of the PT or NTL tissues were used. For assessing the effects of TAMs induced in vitro, the THP-1 cells co-cultured with HCC cells or primary *MMP9*^+^ TAMs sorted from HCC tumors were used. After 24–36 h of incubation, cells were fixed in 3.7% paraformaldehyde in phosphate-buffered saline (PBS). The abilities of cells migration and invasion were then quantified by counting the number of HCCLM3 or Huh7 cells (green) that were on the underside of the filter in five fields under a 10× objective lens and imaged using the SPOT imaging software (Nikon, Japan). The assays were performed for at least three times, each with at least three biological replicates.

### Tube formation assays

To assess the tube formation ability of HUVECs, the 15-well µ-Slide Angiogenesis plates (Ibidi, Germany) were coated with 10 µL Matrigel and were allowed to polymerize for at least 30 min before use. HUVECs were stained with CellTracker Green CMFDA (Invitrogen, USA) for 30 min and washed three times with PBS. Then, approximately 1 × 10^4^ stained HUVECs together with 1 × 10^4^ TAMs in 50 μL medium were seeded into each well on the plate. For assessing the effects of TAMs from clinical samples, TAMs sorted from the single-cell suspensions of PT or NTL tissues were used. For assessing the effects of TAMs induced in vitro, the THP-1 macrophages co-cultured with HCC cells or primary *MMP9*^+^ TAMs sorted from HCC tumors were used. After incubation of 6 h at 37 °C, HUVECs (green) were imaged at ×10 magnification on a TE-2000U inverted microscope (Nikon, Japan), and the total number of tubes in each well were counted as a measurement of the ability of tube formation. The assays were performed for at least three times, each with at least three biological replicates.

### Single-cell gene expression quantification

The Cell Ranger software (version 2.2.0; 10× Genomics, USA) was used to perform sample demultiplexing, barcode processing and single-cell 3’ counting. The *mkfastq* function in Cell Ranger was used to demultiplex the raw base calling files from the sequencer into the sample-specific fastq files. Then, the fastq files for each sample were processed with the *count* function in Cell Ranger, which was used to align the reads to human genome (build hg38) and quantify the gene expression levels in single cells.

### Quality control and batch correction

To filter out low-quality cells and doublets (two cells encapsulated in a single droplet), for each sample, the cells that had either fewer than 200 unique molecular identifiers (UMIs), or over 8000 or below 200 expressed genes, were removed. To filter out dead or dying cells, the cells that had over 10% UMIs derived from mitochondrial genome were further removed. This resulted in a total of 71,915 high-quality single-cell transcriptomes in all samples.

To merge samples across the tissues or patients, we run a canonical correlation analysis (CCA) for batch correction using the *RunMultiCCA* function in R package Seurat (v2). To calculate the canonical correlation vectors (CCVs), the variably expressed genes were selected for each sample as having a normalized expression between 0.125 and 3, and a quantile-normalized variance exceeding 0.5, and then combined across all samples. The resulting 2,773 non-redundant variable genes were summarized by CCA, and the first 15 CCVs were aligned to combine raw gene expression matrices generated per sample. The aligned CCVs were also used for T-distributed Stochastic Neighbor Embedding (tSNE) dimensionality reduction using the *RunTSNE* function in Seurat. Besides the CCA, we also performed the mutual nearest neighbor (MNN)-based and anchor-based batch correction approaches. For MNN-based correction, all the cells across all samples were projected into the low-dimensional space defined by principal component analysis (PCA). Identification of MNNs and calculation of correction vectors were then performed in this low-dimensional space using the *fastMNN* function in R package Scran^47^ with the default number of nearest neighbors. The low-dimensional corrected coordinates were then used for further cell clustering and tSNE dimensionality reduction. For anchor-based correction, the cell pairwise correspondences between single cells across datasets, termed “anchors”, were calculated based on the 2,773 non-redundant variable genes using the *FindIntegrationAnchors* function in Seurat (v3) with default parameters. The expression levels of these variable genes in each sample were corrected using the generated anchors and combined into a single Seurat object.

### Cells clustering

For cell clustering, we used the *FindClusters* function in Seurat (v2), which implements shared nearest neighbor (SNN) modularity optimization-based clustering algorithm. A total of 26–61 clusters were identified using the 30 aligned CCVs, with resolution ranging from 1 to 4. A resolution of 3 was chosen for the analysis and a final of 53 clusters were obtained. To evaluate the effect of cell numbers on clustering results, we iteratively repeated the clustering analysis after down-sampling the data to 1/2, 1/3, 1/4 and 1/5 of all cells. For each down-sampling, 100 replicates were performed. Each down-sampled dataset was used for clustering analysis by *FindClusters*, and the resulting cluster labels were compared with our benchmark labels, as obtained from the whole dataset analysis, using the normalized mutual information (NMI) index^48^. A higher NMI index means more accurate cluster assignment in the down-sampled dataset. Additionally, we performed leave-one-patient-out analysis similar to the down-sampling assessment above. In this analysis, we hold out one patient at a time and compute the NMI index between the clustering labels in the other nine patients and benchmark labels. As expected, this NMI index slightly drops for smaller clusters biased to one patient, but is otherwise highly robust and stable (Supplementary Fig. 1d). Among the 53 cell clusters, 13 clusters are specifically associated to individual patients (>60% cells from a single patient) and are therefore termed as the patient-specific clusters. The other 40 clusters are shared across multiple patients and are termed as the patient-shared clusters.

### Identification of marker genes for cell clusters

To identify the marker genes for each one of those 53 cell clusters, we contrasted cells from a cluster to all the other cells of that cluster using the *FindMarkers* function of Seurat, which identifies differentially expressed genes between two groups of cells using a Wilcoxon rank-sum test. *P* values were then corrected using Bonferroni correction based on the total number of genes in the dataset. Marker genes were required to have an adjusted *P* value < 0.01, an average expression level in that cluster that was at least 2-fold higher than the average expression level in the other clusters, and a detectable percentage in that cluster at least 20% higher than in the other clusters.

### Similarity measurement of cell clusters

We used two different methods to evaluate the cluster similarities. For datasets with raw gene expression matrix available, we adopted a logistic regression model previously used by a pan-cancer scRNA-seq study^8^ (related to Supplementary Figs. 4a and 6c, d). For datasets with only the fold-change (FC) of markers available, we developed a weighted similarity scoring method by multiplying the FC of the shared markers between each pair of clusters respectively and then sum them up (related to Supplementary Fig. 2c).

### CNV estimation for single cells

We used CONICS^49^ to infer large-scale copy number variations (CNVs) from our scRNA-seq data. To infer the copy number status of each cell, the *CONICSmat* module in CONICS was used to fit a two component Gaussian Mixture Model for each chromosomal region (Supplementary Fig. 11c). The mixture model was fitted to the average gene expression level of genes within a chromosome, across all cells. Cells with a deletion at a specific region will show an average lower expression level from that region than cells without the deletion. The posterior probabilities for each cell belonging to one of the components were then used to construct a heatmap that visualizes the copy number status of each cell.

### SCENIC analysis

SCENIC was used to identify the shared regulatory networks by utilizing the putative regulatory binding sites found in promoter regions^20^. To investigate the transcription factor (TF) activity in single cells, SCENIC analysis was run using the pySCENIC and GRNboost2 packages. The required databases for running SCENIC, including the TF database (cisTarget.hg38.mc9nr.feather) and motif annotation database (hgnc.v9.m0.001), were downloaded from the pySCENIC website (https://github.com/aertslab/pySCENIC). The input matrix of pySCENIC was the normalized expression matrix output from Seurat, and the activity of a TF was measured as the Area Under the recovery Curve (AUC) of the genes that are regulated by this TF. To get differentially activated TFs between each two types of cells, the R package limma^50^ was used to fit TF-wise linear models and implements empirical Bayes moderated *t*-statistics to determine the statistical significance (Benjamini-Hochberg-adjusted *P* value <0.01 and *t*-statistics >30). Results of these linear models were visualized using the bar plots or heatmaps based on the *t*-statistics.

### Cell subtype deconvolution from bulk RNA-seq

To assess the function of cell subtypes in larger compendiums of samples, we assessed their composition in bulk RNA-seq data from The Cancer Genome Atlas (TCGA)-liver hepatocellular carcinoma (LIHC) (*n* = 369)^51^ and the Fudan HCC cohorts (*n* = 225)^52^. We assumed that only the abundance of patient-shared cell subtypes could be robustly inferred across samples, so the patient-specific subtypes were excluded from this analysis. The abundance of a cell subtype in a tumor or non-tumor liver tissue was estimated by the sum of log-transformed TPM of all its marker genes in the bulk RNA-seq. The abundance of exhausted T cells was estimated using a set of canonical T cell exhaustion markers, including *CTLA4*, *PDCD1*, *LAG3*, *CD27*, *CD52* and *ICOS*. We normalized the abundance of a cell subtype by dividing its abundance with the sum of the abundance of all patient-shared immune and stromal cell types identified in the HCC single-cell dataset. To assess the clinical relevance of these cell subtypes, we applied a Cox proportional hazards model implemented in the R *survival* package.

### Identification of TLS^high^ and TLS^low^ tumors from bulk RNA-seq

We used an approach similar with cell subtype deconvolution to infer the tertiary lymphoid structure (TLS) abundances of tumors from bulk RNA-seq data. Two different TLS signatures that have been widely used for TLS detection in various tumors were adopted for this analysis: a 9-gene signature^53^ and a 12-gene signature^54^. The 9-gene signature includes a set of immune cell-specific genes: *CD79B*, *CD1D*, *CCR6*, *LAT*, *SKAP1*, *CETP*, *EIF1AY*, *RBP5* and *PTGDS*, while the 12-gene signature includes a set of genes encoding chemokines: *CCL2*, *CCL3*, *CCL4*, *CCL5*, *CCL8*, *CCL18*, *CCL19*, *CCL21*, *CXCL9*, *CXCL10*, *CXCL11* and *CXCL13*. The abundance of TLS in a tumor was estimated by the sum of log-transformed TPM of two sets of signature genes, respectively, and the tumors in the TCGA-LIHC and Fudan cohorts, respectively, were divided into two groups: TLS^high^ and TLS^low^ using the median split. To assess the clinical relevance of TLS abundance, we applied a Cox proportional hazards model implemented in the R *survival* package.

### Tumor microenvironment classification

We partitioned the HCC patients using the *k*-means clustering method based on the absolute or normalized abundance of non-malignant cell subtypes inferred from bulk RNA-seq. We found the patient groups divided based on the absolute abundances of cell subtypes do not show significant difference in patient prognosis (*P* = 0.3) (Supplementary Figs. 15a, b). Correlation analyses of absolute cellular abundances showed that most non-malignant cell subtypes are highly correlated with one another across patients (Supplementary Fig. 15c). We showed that consistent with previous study^55^, the averaged absolute abundance of non-malignant cell clusters is also strongly negatively correlated with tumor purity (*R* = –0.70, *P* = 1.4 × 10^–53^, Spearman’s ρ test; Supplementary Fig. 15d), which estimates the relative proportion of malignant and non-malignant cell clusters in a tumor. As a result, we chose to partition the HCC patients based on the normalized abundance of non-malignant cell subtypes.

### Gene set variation analysis

Pathway analyses were predominantly performed on the 50 hallmark pathways described in the molecular signature database (v7) of gene set enrichment analysis (GSEA)^56^. To reduce the pathway overlaps and redundancies, each gene set associated with a pathway was trimmed to only contain unique genes, and all genes associated with two or more pathways were removed. Most gene sets retained >70% of their associated genes. Then, we applied the gene set variation analysis (GSVA)^57^ with standard settings, as implemented in the R *GSVA* package, to assign the pathway activity estimates to individual cells.

### StemID-based cellular lineage analysis

StemID2 infers the links between cell clusters which are more populated with intermediate single-cell transcriptomes than expected by chance^19^. For a separate analysis of the macrophage population, all the cells from clusters C5, C6, C16, C21, C23 and C26 were extracted and analyzed using StemID2 as implemented in the R *RaceID* package. The entropy of each cell type, which is required to compute the StemID2 score, was computed using the *compentropy* function with default parameters. The dimensionality reduction and the calculation of the projections of each cell onto all inter-cluster links are performed by the *projcells* function with default parameters. Finally, the lineage graph was assembled based on the cell projections onto inter-cluster links using the *lineagegraph* function with default parameters. The significance of links of the lineage tree was determined by circumvent time-intense randomizations of the lineage tree.

### RNA velocity-based cell fate tracing

RNA velocity infers the precursor-progeny cell dynamics between subpopulations by distinguishing between unspliced and spliced mRNAs in scRNA-seq data^58^. To perform the RNA velocity analysis, the spliced reads and unspliced reads were recounted by the velocyto python package based on previous aligned bam files of scRNA-seq data. The calculation of RNA velocity values for each gene in each cell and embedding RNA velocity vector to low-dimension space were done using velocyto.R and *destiny* R package. We estimated the destination of a cell by identifying the highest correlation value. Then, Fisher’s exact test was performed on 2 × 2 cluster-by-cluster or cluster-by-tissue contingency tables to test the fate destinations of interested cell clusters. To infer the differential directions of macrophages, we first constructed partition-based graph abstraction for macrophage, and then oriented edges among cell populations using the RNA velocity information.

### Cell-cell interaction analysis

The cell-cell interaction analysis was based on the expression of specific ligands (Ls) and receptors (Rs). A total of 1169 literature-supported and manually curated ligand-receptor (L-R) interactions were collected from the Fantom5 and CellPhoneDB databases^59,60^. Cell clusters that had at least 5 cells and occupied 10% of immune cells from either primary tumor or non-tumor liver tissues were considered. We estimated the potential interaction between two cell clusters mediated by a specific L-R pair by the product of the average expression levels of the ligand in one cell cluster and the corresponding receptor in the other cluster. To examine the statistical significance of the estimated interaction intensity, permutations were applied on the cell cluster tags of individual cells for 1000 times, and the *P* value was estimated by the number of permutations that had interaction intensity larger than the real value. Adjusted *P* value by Bonferroni correction was calculated for multiple testing correction across the hundreds of L-R pairs. If a pair of ligand and receptor had a value of interaction intensity larger than 1, and an adjusted *P* value less than 0.01 between two cell clusters, we defined this L-R pair as a potential molecular axis mediating interactions between the two cell clusters. For a given pair of ligand and receptor, cell clusters with the average expression level of either the ligand or the receptor less than 1 (log_2_(Normalized Counts) <1) were filtered. The cellular communication intensity between two cell types was defined as the number of significant L-R interactions between them weighted by the number of cells in the corresponding tissue.

### Bulk RNA sequencing and data processing

Total RNAs were extracted from the non-co-cultured and co-cultured THP-1 cells using the RNeasy Mini Kit (QIAGEN, Germany) according to the manufacturer’s instructions. The qualities of total RNAs were measured by spectrophotometer (NanoDrop, Thermo Fisher, USA). Libraries were constructed using the NEBNext Poly(A) mRNA Magnetic Isolation Module kit (NEB, USA) and NEBNext Ultra RNA Library Prep Kit for Illumina Paired-end Multiplexed Sequencing Library (NEB, USA). Samples were sequenced on the Illumina Hiseq 4000 sequencer with 150 bp paired-end reads. The Bulk RNA-seq data were processed using the STAR-rsem pipeline. Read counts per gene were normalized to gene length and to the total read counts, and directly compared to the log-transformed UMI count per gene for the single-cell samples using the Pearson correlation analysis.

### Data visualization

Boxplots are defined as follows: the middle line corresponds to the median; the lower and upper hinges correspond to first and third quartiles, respectively; the upper whisker extends from the hinge to the largest value no further than 1.5× the inter-quartile range (or the distance between the first and third quartiles) from the hinge and the lower whisker extends from the hinge to the smallest value at most 1.5× the inter-quartile range of the hinge.

### Reporting summary

Further information on research design is available in the Nature Research Reporting Summary linked to this article.

## Supplementary information

Supplementary Information

Description of Additional Supplementary Files

Supplementary Datasets 1–11

Reporting Summary

## Data Availability

The raw data of single-cell RNA sequencing generated in this study have been deposited in the European Genome-phenome Archive (EGA) database under accession code EGAC00001001616. Because all data submitted to the EGA must be subject to controlled access as defined by the original informed consents, researchers have to propose a scientific hypothesis to require access by contacting Prof. Gangqiao Zhou. The processed gene expression data of single-cell RNA sequencing is available at the Gene Expression Omnibus (GEO) database under accession code GSE149614. The raw and processed gene expression data of bulk RNA sequencing is available at the GEO database under accession code GSE168922. The L-R data used in this study are available in the Fantom5 and CellPhoneDB databases via https://fantom.gsc.riken.jp/5/suppl/Ramilowski_et_al_2015/ and https://www.cellphonedb.org/downloads. Analyzing, visualizing, and downloading of the single-cell transcriptome data is available at http://omic.tech/scrna-hcc/. Source data are provided with this paper.

## Source data

Source Data
