# Supplementary material for: A single-cell atlas of the multicellular ecosystem of primary and metastatic hepatocellular carcinoma
Source: Nat Commun. 2022 Aug 6;13:4594. doi: 10.1038/s41467-022-32283-3 (PMC9357016; doi:10.1038/s41467-022-32283-3)
Supplement: Supplementary file 1 — Supplementary Information [file 41467_2022_32283_MOESM1_ESM.pdf]

A Single-Cell Atlas of the Multicellular Ecosystem of Primary and Metastatic Hepatocellular Carcinoma

This PDF file includes:

Supplementary Figures 1-15 (legends included)

a

Patients	NTL	PT	PVTT	MLN	Viral infection	Stages
HCC01	No	Yes	No	No	HBV	I
HCC02	No	Yes	No	No	HBV	I
HCC03	Yes	Yes	No	No	HCV	I
HCC04	Yes	Yes	No	No	HCV	II
HCC05	Yes	Yes	No	No	None	IIIA
HCC06	Yes	Yes	No	No	HBV	IIIA
HCC07	Yes	Yes	Yes	No	None	IIIB
HCC08	Yes	Yes	Yes	No	None	IIIB
HCC09	Yes	Yes	No	No	HBV	IV
HCC10	Yes	Yes	No	Yes	HBV	IV

b

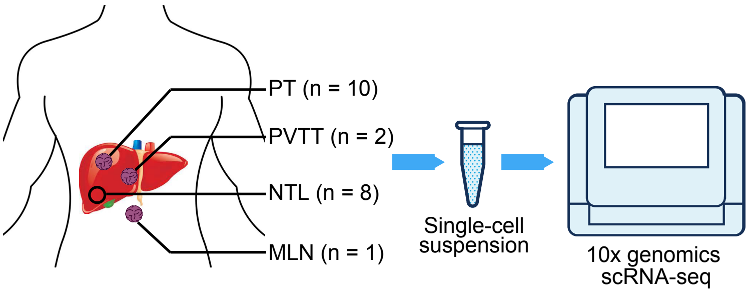

c

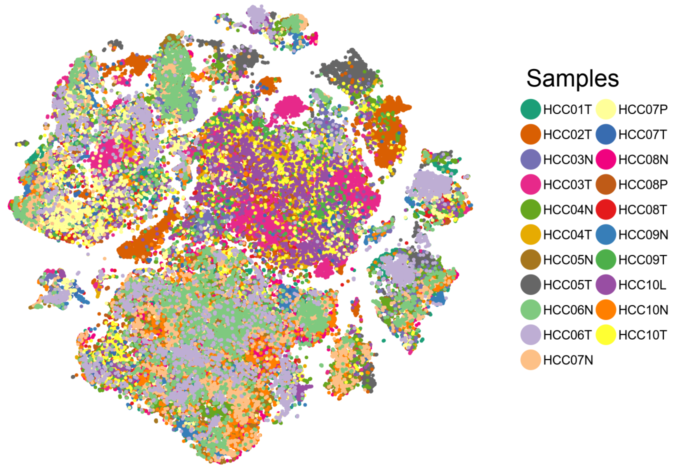

d

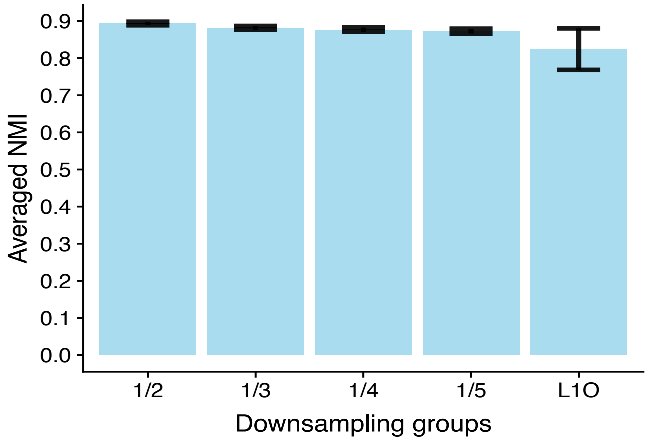

Supplementary Figure 1 | Single-cell RNA sequencing of primary and metastatic HCCs, and non-tumor liver tissues.

a Summary of the patients and their sequenced samples. More details are provided in Supplementary Data 1. NTL, non-tumor liver; PT, primary tumor; PVTT, portal vein tumor thrombus; MLN, metastatic lymph node. **b** Graphical workflow of experiment strategy. **c** The T-distributed Stochastic Neighbor Embedding (tSNE) plot of all cells with mutual nearest neighbor (MNN)-based batch correction, colored by samples. **d** The normalized mutual information (NMI) index between the clustering results of benchmark dataset and 100 times down-sampling datasets, as well as leave-one-patient-out datasets (n = 100 randomly down-sampling replicates for each group). 1/2, 1/3, 1/4 and 1/5 indicate the fractions of down-sampling. L1O, leave-one-patient-out. Error bars, mean ± sd. Source data are provided as a Source Data file.

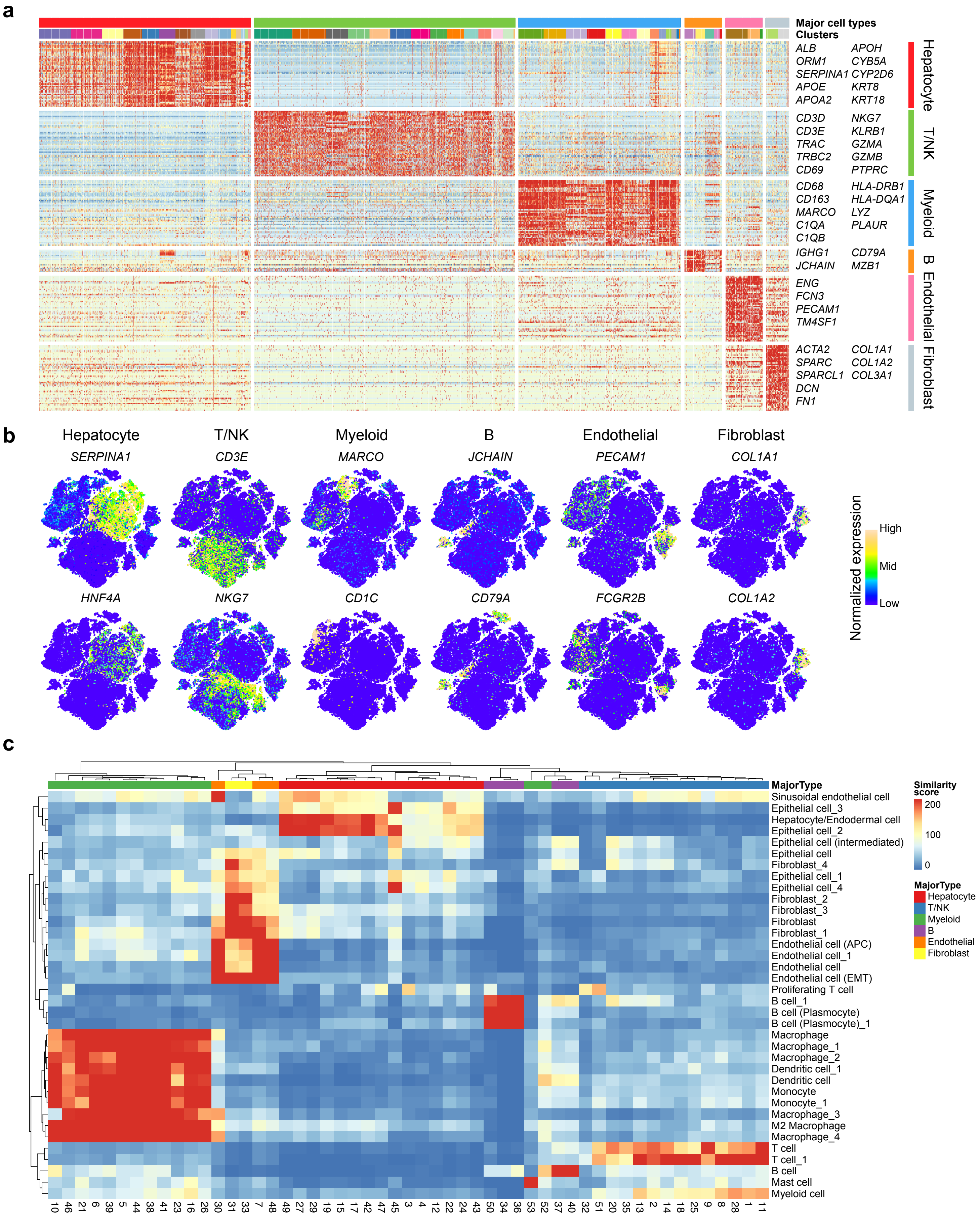
a
Major cell types
Clusters
ALB APOH
ORM1 CYB5A
SERPINA1 CYP2D6
APOE KRT8
APOA2 KRT18
CD3D NKG7
CD3E KLRB1
TRAC GZMA
TRBC2 GZMB
CD69 PTPRC
CD68 HLA-DRB1
CD163 HLA-DQA1
MARCO LYZ
C1QA PLAUR
C1QB
IGHG1 CD79A
JCHAIN MZB1
ENG
FCN3
PECAM1
TM4SF1
ACTA2 COL1A1
SPARC COL1A2
SPARCL1 COL3A1
DCN
FN1
Hepatocyte
T/NK
Myeloid
B
Endothelial
Fibroblast
b
Hepatocyte
T/NK
Myeloid
B
Endothelial
Fibroblast
SERPINA1
CD3E
MARCO
JCHAIN
PECAM1
COL1A1
HNF4A
NKG7
CD1C
CD79A
FCGR2B
COL1A2
Normalized expression
High
Mid
Low
c
MajorType
Sinusoidal endothelial cell
Epithelial cell_3
Hepatocyte/Endodermal cell
Epithelial cell_2
Epithelial cell (intermediated)
Epithelial cell
Fibroblast_4
Epithelial cell_1
Epithelial cell_4
Fibroblast_2
Fibroblast_3
Fibroblast
Fibroblast_1
Endothelial cell (APC)
Endothelial cell_1
Endothelial cell
Endothelial cell (EMT)
Proliferating T cell
B cell_1
B cell (Plasmocyte)
B cell (Plasmocyte)_1
Macrophage
Macrophage_1
Macrophage_2
Dendritic cell_1
Dendritic cell
Monocyte
Monocyte_1
Macrophage_3
M2 Macrophage
Macrophage_4
T cell
T cell_1
B cell
Mast cell
Myeloid cell
10 46 21 6 39 5 44 38 41 23 16 26 30 31 33 7 48 49 27 29 19 15 17 42 47 45 3 4 12 22 24 43 50 34 36 53 52 37 40 32 51 20 35 13 2 14 18 25 9 8 28 1 11
Similarity score
200
100
0
MajorType
Hepatocyte
T/NK
Myeloid
B
Endothelial
Fibroblast

Supplementary Figure 2 | Cell type annotation of HCC cellular microenvironment.

a Heatmap showing the expression profiles of upregulated genes in six major cell types. Canonical marker genes are listed at the right side of the map. Rows represent the upregulated genes. Columns represent the individual cells. **b** The tSNE plots showing the expression levels of signature genes of six major cell types, colored by gene expression. Mid, middle. **c** Heatmap showing the similarity scores between 53 cell clusters and annotated cell types in the Human Cell Landscape (HCL) project. Only liver- or HCC-related cell types were included in the analysis. Source data are provided as a Source Data file.

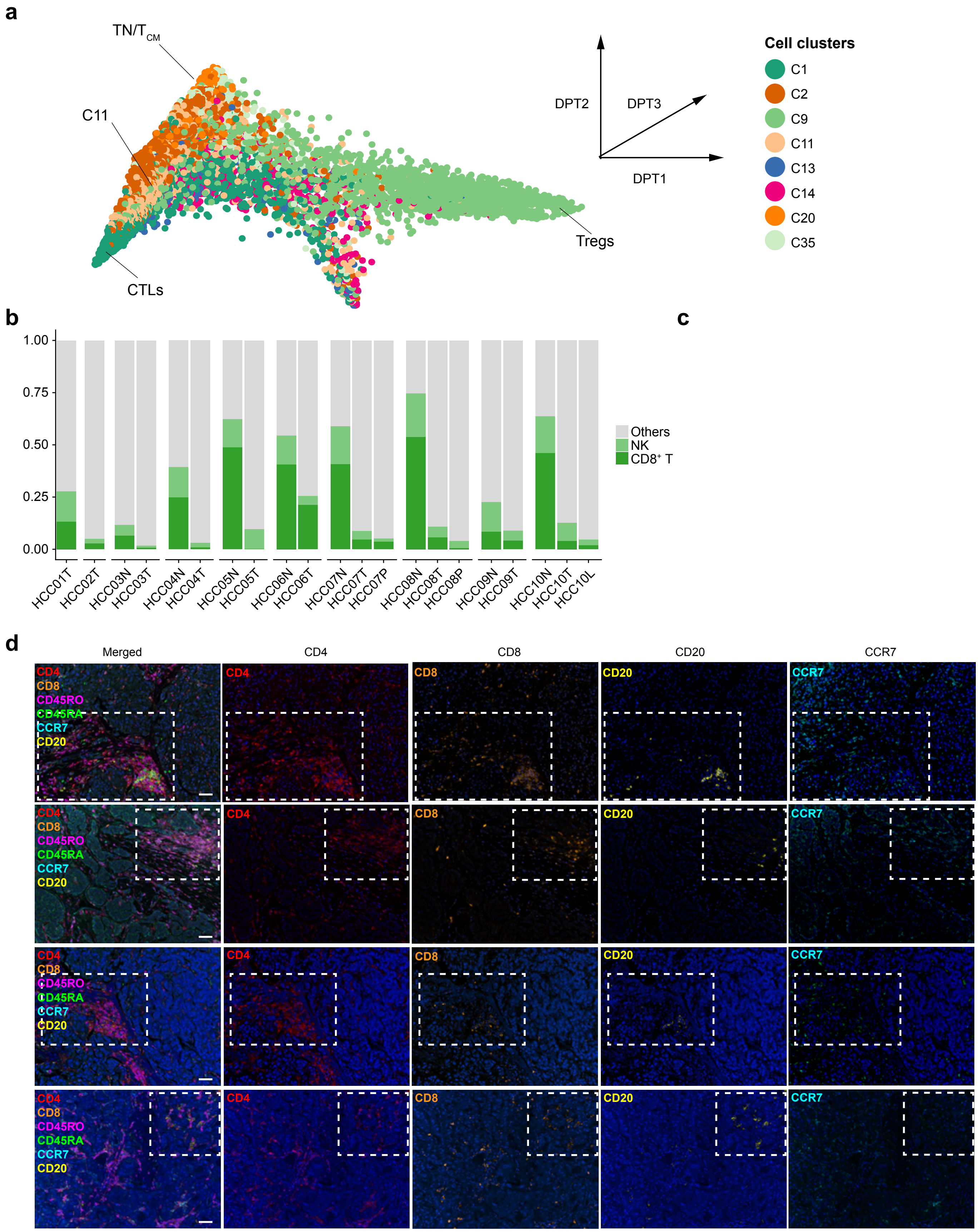
a
TN/TCM
C11
CTLs
Tregs
DPT2
DPT3
DPT1
Cell clusters
C1
C2
C9
C11
C13
C14
C20
C35
b
1.00
0.75
0.50
0.25
0.00
HCC01T
HCC02T
HCC03N
HCC03T
HCC04N
HCC04T
HCC05N
HCC05T
HCC06N
HCC06T
HCC07N
HCC07T
HCC07P
HCC08N
HCC08T
HCC08P
HCC09N
HCC09T
HCC10N
HCC10T
HCC10L
Others
NK
CD8+ T
c
d
Merged
CD4
CD8
CD20
CCR7
CD4
CD8
CD45RO
CD45RA
CCR7
CD20

Supplementary Figure 3 | Characterization of T cells in the cellular microenvironment of HCC.

a Diffusion maps of T cells showing C11 cluster is related to an intermediate state between the TN/T_{CM} cells (C20 and C35) and cytotoxic T lymphocytes (CTLs) (C1). Three diffusion components (DCs) are indicated by arrows. Cells of different T cell clusters are marked by colors. **b** The fractions of $CD8^{+}$ T and natural killer (NK) cells in primary/metastatic tumor and non-tumor liver tissues. **c** Density of $CD4^{+}CCR7^{+}CD45RA^{+}$ naïve T (TN) and $CD4^{+}CCR7^{+}$ $CD45RO^{+}$ central memory T (T_{CM}) cells in paired primary tumor and non-tumor liver tissues (n = 9 HCC independent patients). Among the nine HCC patients, six patients (HCC03, HCC04, HCC05, HCC06, HCC08 and HCC09) have been performed scRNA-seq on their tissue samples, and three (HCC11, HCC12 and HCC13) were the additional recruited HCC patients. Cell density was obtained using InForm (PerkinElmer) software. Boxplot elements are defined in the Methods section (section on data visualization). **d** Multicolor immunohistochemistry (IHC) staining to validate the enrichment of $CD4^{+}$ T_{CM} (center) in primary tumors, exemplified by patients HCC03, HCC04, HCC05, HCC06 and HCC08, respectively. The scale bars represent 50 μm. Source data are provided as a Source Data file.

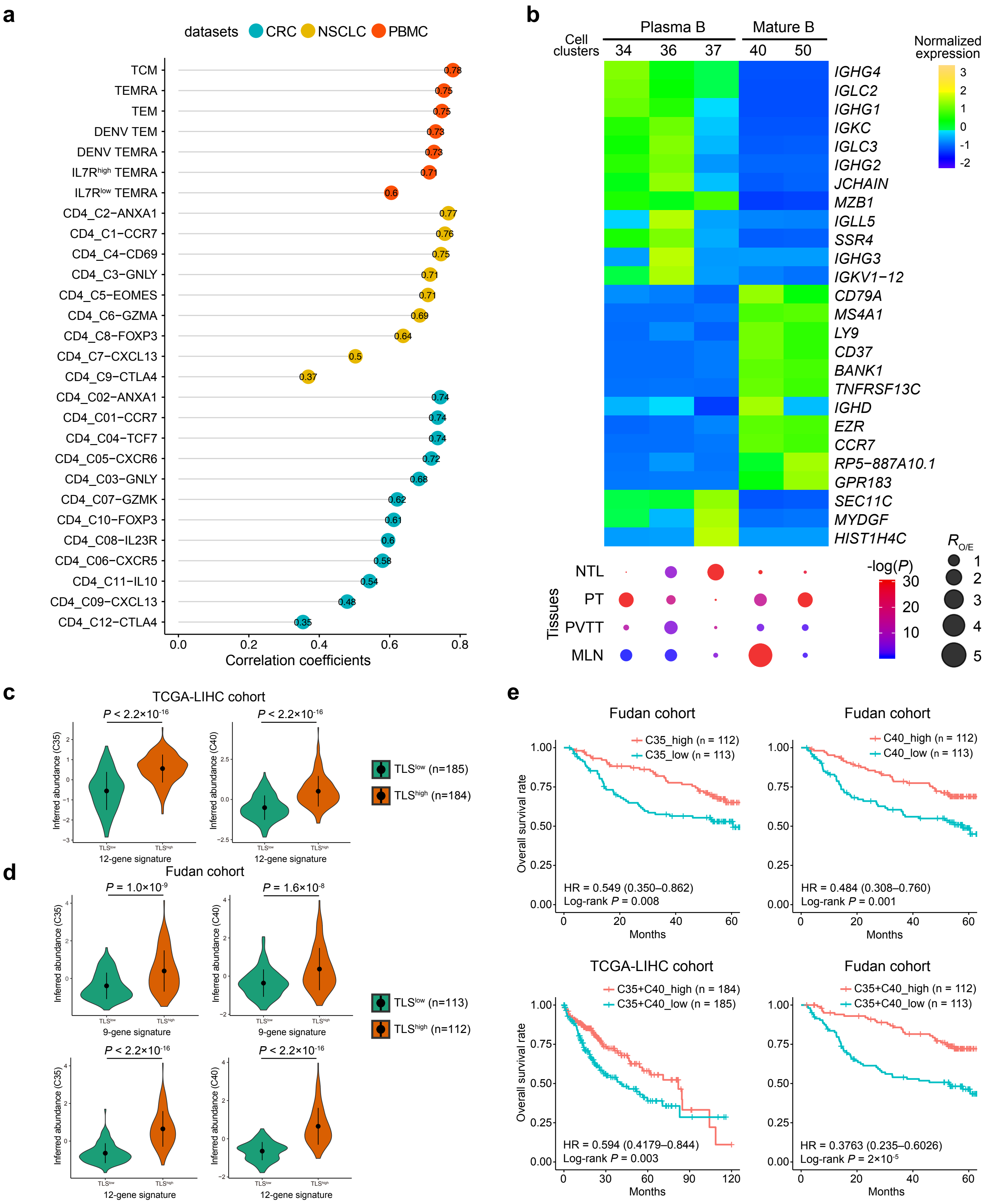

a
datasets CRC NSCLC PBMC
Correlation coefficients
b
Plasma B
Mature B
Cell clusters
Normalized expression
Tissues
NTL
PT
PVTT
MLN
-log(P)
c
TCGA-LIHC cohort
12-gene signature
d
Fudan cohort
9-gene signature
12-gene signature
e
Fudan cohort
TCGA-LIHC cohort
Overall survival rate
Months
HR = 0.549 (0.350–0.862)
Log-rank P = 0.008
HR = 0.484 (0.308–0.760)
Log-rank P = 0.001
HR = 0.594 (0.4179–0.844)
Log-rank P = 0.003
HR = 0.3763 (0.235–0.6026)

Supplementary Figure 4 | Identification of intratumoral central memory T cells by multicolor immunohistochemistry assays.

a Cluster similarity between CD4$^+$ central memory T (T_{CM}) cells in C35 and T cell subtypes identified in peripheral blood mononuclear cells (PBMC), non-small-cell lung cancer (NSCLC) and colorectal cancer (CRC). **b** Heatmap (top) showing the expression profiles of canonical marker genes of B cell subtypes and dotplot (bottom) showing their tissue preferences. Dot size indicates the ratios of the observed *versus* expected cell numbers ($R_{O/E}$); Dot color indicates the log-transformed P values determined by two-sided Chi-squared test. **c** and **d** Differential abundances of CD4$^+$ central memory T (T_{CM}; C35) and CD20$^+$ B cells (C40) between the TLS^{low} and TLS^{high} HCC tumors. The bulk RNA-seq datasets of HCC tumors from The Cancer Genome Atlas (TCGA)-liver hepatocellular carcinoma (LIHC) cohort (b) (n = 369) and the Fudan HCC cohort (c) (n = 225) were used in the analyses. The HCC tumors were classified as TLS^{low} or TLS^{high} tumors based on a 9-gene TLS signature and a 12-gene TLS signature using median split, respectively. The abundances of cell subtypes were inferred from the bulk RNA-seq data using the signature genes of respective subtype. The statistical significance was determined by unpaired two-tailed t test. TLS, tertiary lymphoid structures. Source data are provided as a Source Data file.

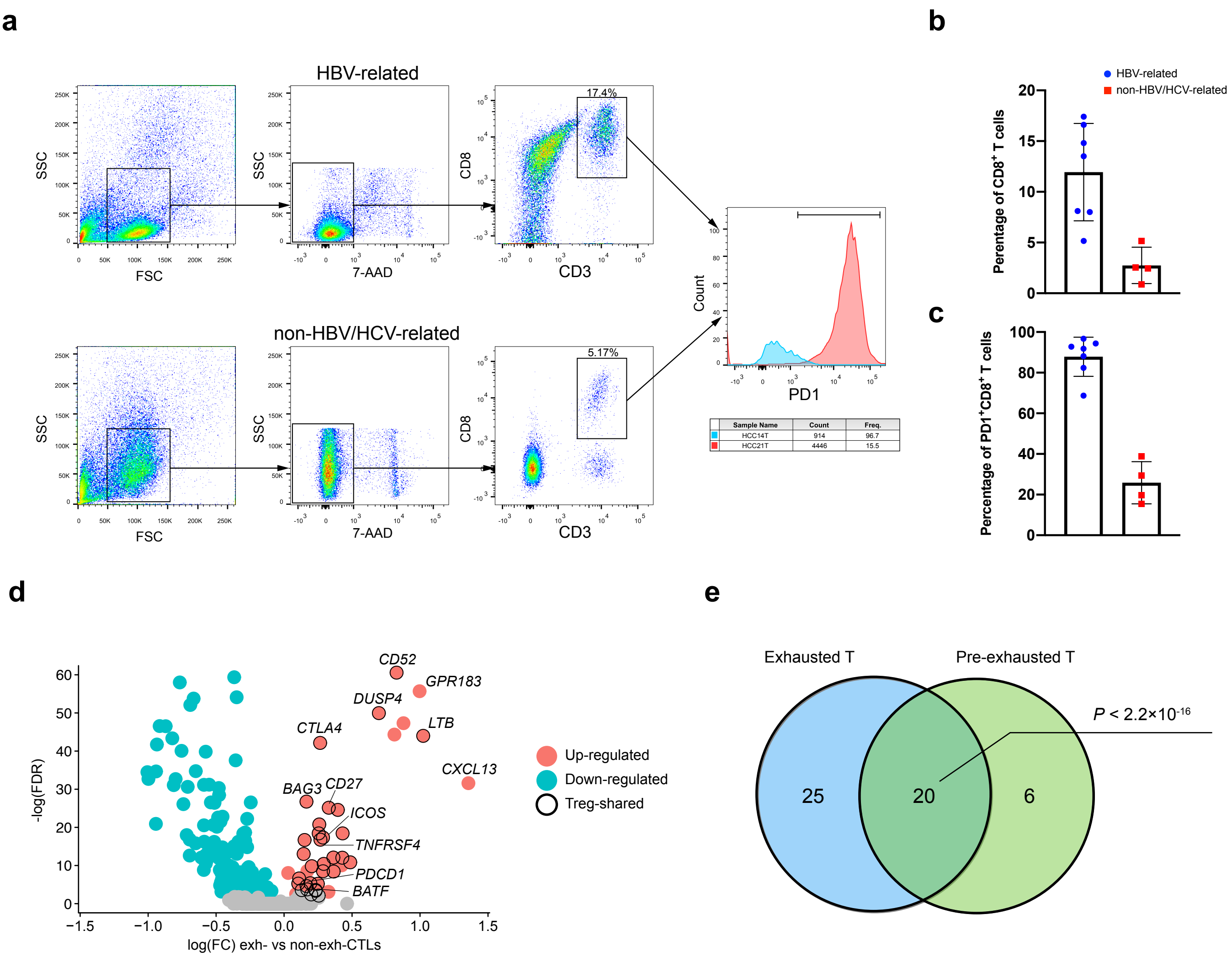
a
HBV-related
non-HBV/HCV-related
SSC
FSC
7-AAD
CD8
CD3
17.4%
5.17%
Count
PD1
Sample Name
Count
Freq.
HCC14T
914
96.7
HCC21T
4446
15.5
b
HBV-related
non-HBV/HCV-related
Perentage of CD8+ T cells
c
Percentage of PD1+CD8+ T cells
d
-log(FDR)
log(FC) exh- vs non-exh-CTLs
CD52
GPR183
DUSP4
LTB
CTLA4
CXCL13
BAG3
CD27
ICOS
TNFRSF4
PDCD1
BATF
Up-regulated
Down-regulated
Treg-shared
e
Exhausted T
Pre-exhausted T
P < 2.2×10-16
25
20
6

Supplementary Figure 5 | T cell exhaustion in HBV-/HCV-related and non-HBV/HCV-related HCC tumors.

a Fluorescence-activated cell sorting (FACS) gating strategy of $PD1^+$ exhausted $CD8^+$ T cells sorted from primary tumor (PT) tissues in HBV-/HCV-related and non-HBV/HCV-related HCC tumors. Percentages of $CD8^+$ T cells in $CD45^+$ immune cells **b** and $PD1^+$ exhausted $CD8^+$ T cells in $CD8^+$ T cells **c** in primary tumor (PT) tissues in HBV-/HCV-related (n = 7 independent HCC patients) and non-HBV/HCV-related HCC tumors (n = 4 independent HCC patients). Error bars, mean ± sd. **d** Up- and down-regulated genes in the exhausted CTLs compared to non-exhausted CTLs. The significantly up-regulated genes are colored by red, the significantly down-regulated genes are colored by blue, and genes without significant expression changes are colored by grey. The genes also up-regulated in Tregs are marked by solid circles. CTLs, cytotoxic T lymphocytes. **e** Venn plot showing that the up-regulated genes (n = 45) in the exhausted CTLs (KM5) and the up-regulated genes (n = 26) in the pre-exhausted CTLs (KM4) are significantly overlapped. The statistical significance was determined by hypergeometric test. Source data are provided as a Source Data file.

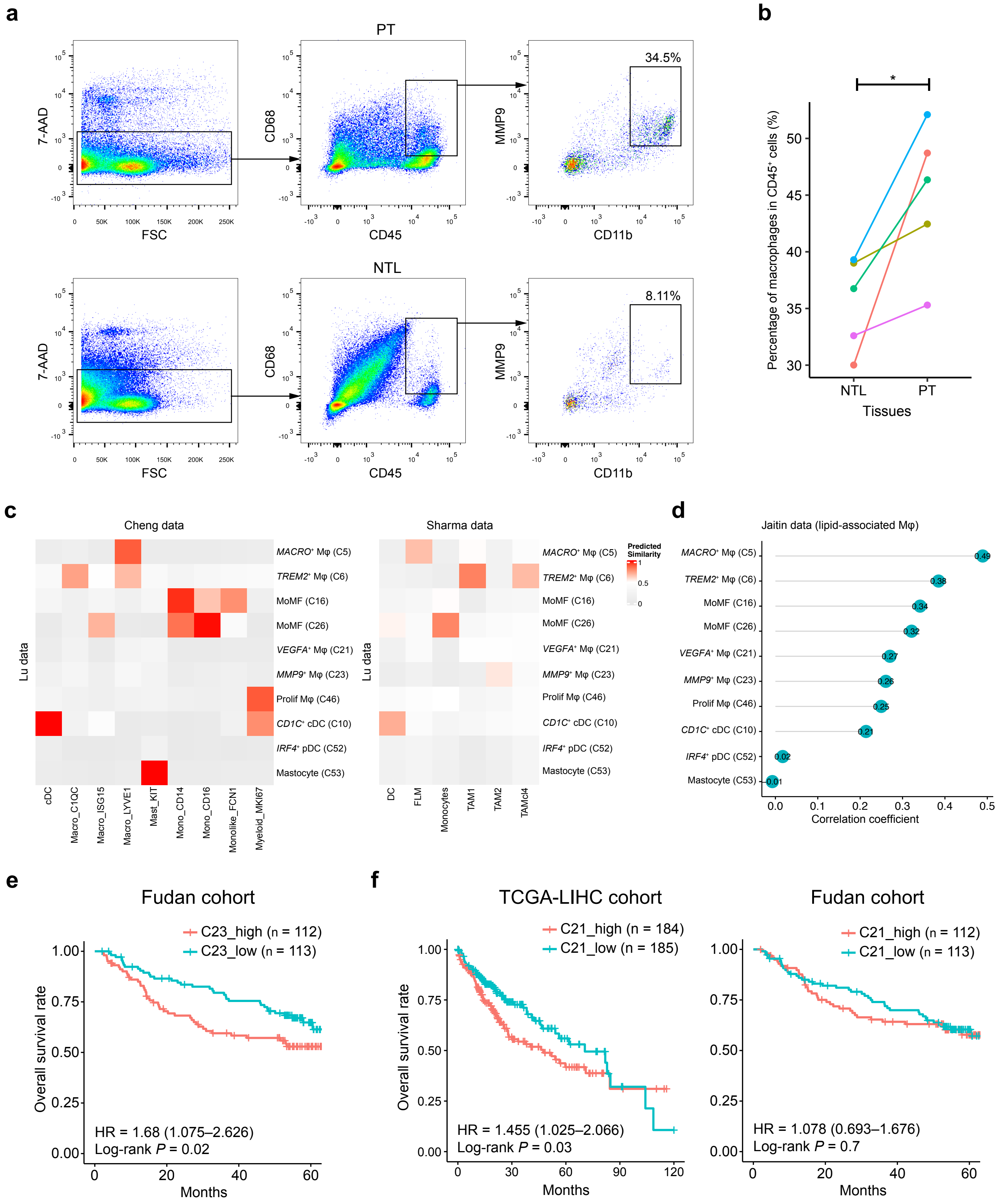

a
PT
NTL
7-AAD
FSC
CD68
CD45
MMP9
CD11b
34.5%
8.11%
b
*
Percentage of macrophages in CD45⁺ cells (%)
NTL
PT
Tissues
c
Cheng data
Sharma data
Lu data
MACRO⁺ Mφ (C5)
TREM2⁺ Mφ (C6)
MoMF (C16)
MoMF (C26)
VEGFA⁺ Mφ (C21)
MMP9⁺ Mφ (C23)
Prolif Mφ (C46)
CD1C⁺ cDC (C10)
IRF4⁺ pDC (C52)
Mastocyte (C53)
cDC
Macro_C1QC
Macro_ISG15
Macro_LYVE1
Mast_KIT
Mono_CD14
Mono_CD16
Monolike_FCN1
Myeloid_MKI67
DC
FLM
Monocytes
TAM1
TAM2
TAMcl4
Predicted Similarity
d
Jaitin data (lipid-associated Mφ)
0.49
0.38
0.34
0.32
0.27
0.26
0.25
0.21
0.02
0.01
Correlation coefficient
e
Fudan cohort
C23_high (n = 112)
C23_low (n = 113)
Overall survival rate
HR = 1.68 (1.075–2.626)
Log-rank P = 0.02
Months
f
TCGA-LIHC cohort
C21_high (n = 184)
C21_low (n = 185)
HR = 1.455 (1.025–2.066)
Log-rank P = 0.03
Fudan cohort
C21_high (n = 112)
C21_low (n = 113)
HR = 1.078 (0.693–1.676)
Log-rank P = 0.7

Supplementary Figure 6 | Characterization of *MMP9*$^+$ tumor-associated macrophages (TAMs) in the HCC cellular microenvironment.

a Fluorescence-activated cell sorting (FACS) gating strategy of *MMP9*$^+$ TAMs from the primary tumor (PT) and non-tumor liver (NTL) tissues of HCC patient. **b** The proportion of CD45$^+$CD68$^+$ macrophages in CD45$^+$ immune cells in primary tumors (PT) is significantly higher than in non-tumor liver (NTL) tissues, measured by FACS in five HCC patients. The statistical significance was determined by two-sided paired Student's *t* test. **c** Heatmaps showing cluster similarities between patient-shared myeloid clusters identified in this study and myeloid cell subtypes identified in Cheng's pan-cancer (left) and Sharma's HCC (right) datasets. **d** Lollipop plot showing similarities between lipid-associated macrophages in Jaitin's study and myeloid clusters identified in this study. **e** Higher abundances of *MMP9*$^+$ TAMs (C23) in tumors predict worse overall survival (OS) rates in HCC patients from the Fudan cohort. Classification of C23_high/C23_low and C21_high/C21_low was performed by median split respectively. Hazard ratio (HR) (with 95% confidence interval in brackets) was calculated using a Cox proportional hazards regression model, and the statistical significance was determined by log-rank test. **f** The associations between the abundances of *VEGFA*$^+$ TAMs (C21) in tumors and the overall survival (OS) rates in HCC patients from both the TCGA-LIHC (left) and the Fudan cohort (right). Source data are provided as a Source Data file.

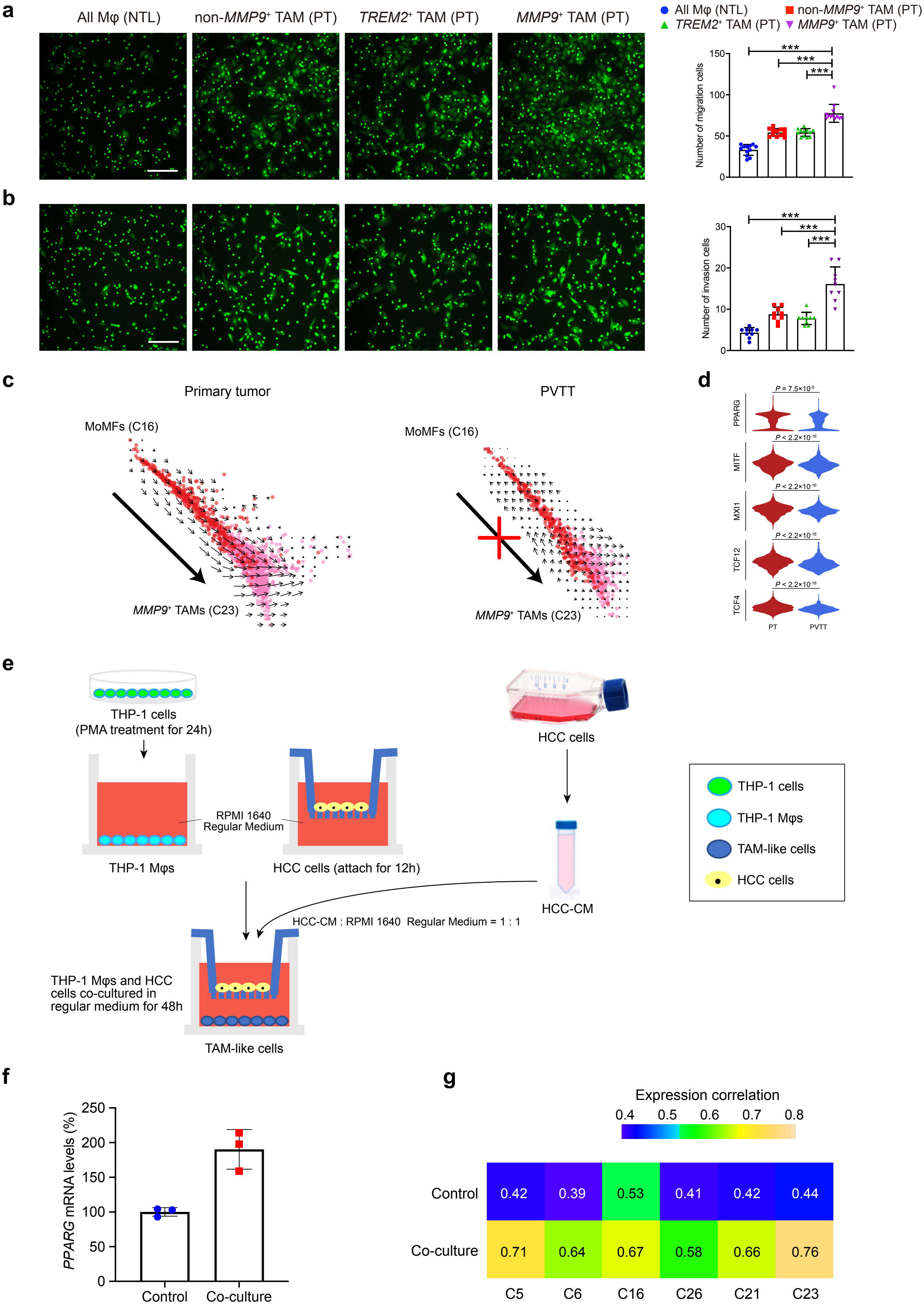
a
All Mφ (NTL)
non-MMP9+ TAM (PT)
TREM2+ TAM (PT)
MMP9+ TAM (PT)
All Mφ (NTL)
non-MMP9+ TAM (PT)
TREM2+ TAM (PT)
MMP9+ TAM (PT)
Number of migration cells
150
100
50
0
***
***
***
b
Number of invasion cells
30
20
10
0
***
***
***
c
Primary tumor
PVTT
MoMFs (C16)
MMP9+ TAMs (C23)
MoMFs (C16)
MMP9+ TAMs (C23)
d
P = 7.5×10-9
P < 2.2×10-16
P < 2.2×10-16
P < 2.2×10-16
P < 2.2×10-16
PPARG
MITF
MXI1
TCF12
TCF4
PT
PVTT
e
THP-1 cells
(PMA treatment for 24h)
HCC cells
RPMI 1640
Regular Medium
THP-1 Mφs
HCC cells (attach for 12h)
HCC-CM
HCC-CM : RPMI 1640 Regular Medium = 1 : 1
THP-1 Mφs and HCC
cells co-cultured in
regular medium for 48h
TAM-like cells
THP-1 cells
THP-1 Mφs
TAM-like cells
HCC cells
f
PPARG mRNA levels (%)
250
200
150
100
50
0
Control
Co-culture
g
Expression correlation
0.4
0.5
0.6
0.7
0.8
Control
Co-culture
0.42
0.39
0.53
0.41
0.42
0.44
0.71
0.64
0.67
0.58
0.66
0.76
C5
C6
C16
C26
C21
C23

Supplementary Figure 7 | *MMP9*$^{+}$ TAMs promote HCC cells invasion and migration.

a and **b** HCCLM3 cells treated with *MMP9*$^{+}$ TAMs isolated from the PT tissues show increased abilities of **d** migration (n = 12 biological replicates) and **e** invasion (n = 9 biological replicates) compared to the control groups treated with the *TREM2*$^{+}$ TAMs, non-*MMP9*$^{+}$ TAMs isolated from the PT tissues and the whole macrophage populations isolated from the NTL tissues, respectively. The scale bars represent 20 μm. Error bars, mean ± sd. The statistical significance was determined by two-sided Student's *t* test. $^{*}P < 0.05$, $^{**}P < 0.01$ and $^{***}P < 0.001$. **c** RNA velocities between the *MMP9*$^{+}$ (C23) and monocyte-derived macrophages (MoMFs) in C16 cluster are visualized on DPT projection in primary tumor (left) and portal vein tumor thrombus (PVTT) (right). **d** The differential activities of *MMP9*$^{+}$ TAM-related TFs in the *MMP9*$^{+}$ TAMs (C23) and MoMFs (C16) between PT and PVTT tissues. Unpaired two-tailed *t* test. **e** The coculture procedures of THP-1 cells with HCC cells. Briefly, THP-1 cells were first induced to differentiate into THP-1 macrophages by PMA treatment before the coculture. THP-1 macrophages were then cocultured with HCC cells to differentiate into TAM-like cells. **f** Expression levels of *PPARG* measured by RT-qPCR in THP-1 macrophages cultured alone (Control), cocultured with HCC cells (Co-culture) (n = 3 biological replicates). Error bars, mean ± sd. **g** The transcriptome-wide gene expression correlations between the *MMP9*$^{+}$ TAMs sorted from PT by FACS and the six patient-shared macrophage clusters (C5, C6, C16, C26, C21 and C23) identified by scRNA-seq in this study. Source data are provided as a Source

Data file.

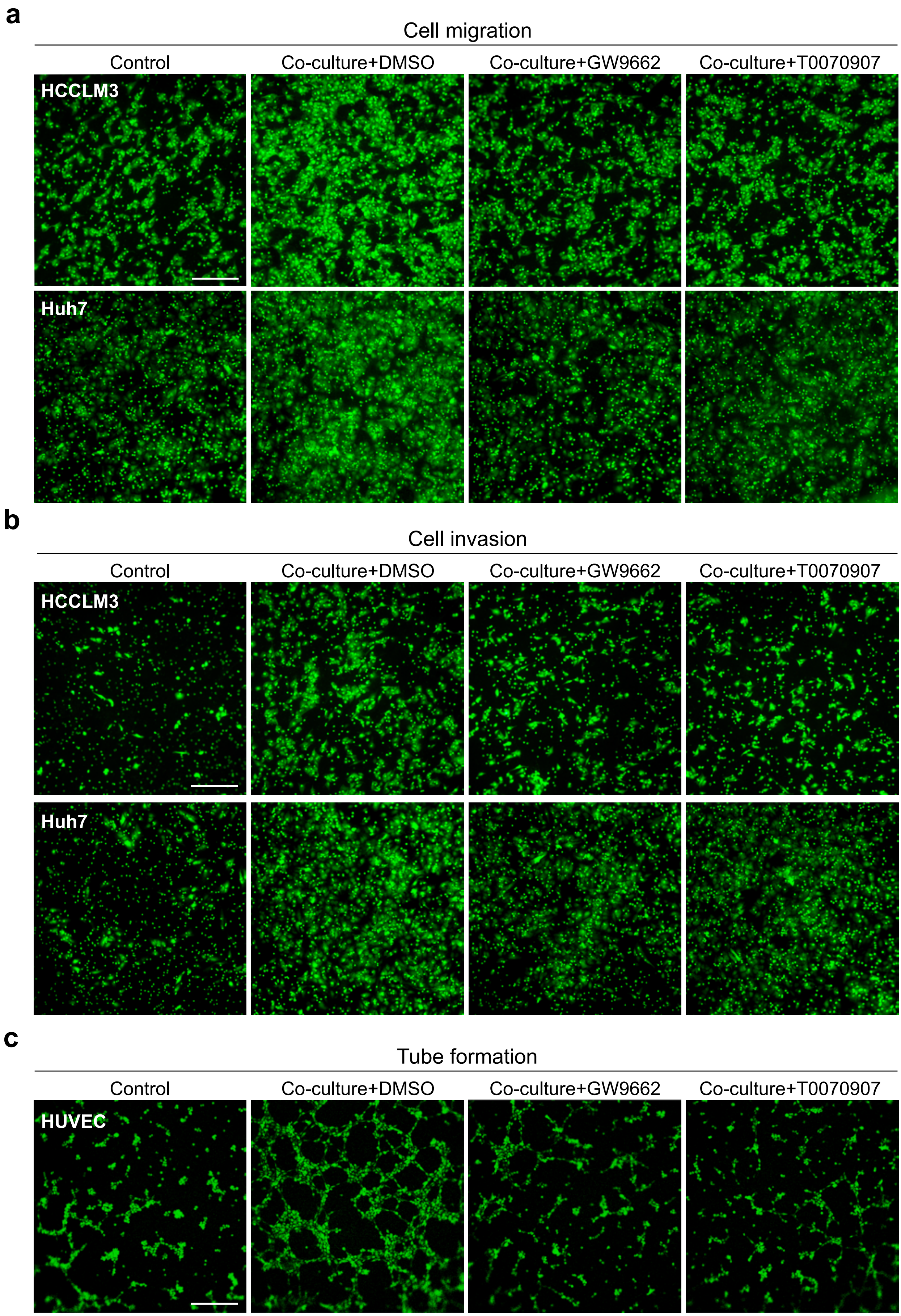
a
Cell migration
Control
Co-culture+DMSO
Co-culture+GW9662
Co-culture+T0070907
HCCLM3
Huh7
b
Cell invasion
Control
Co-culture+DMSO
Co-culture+GW9662
Co-culture+T0070907
HCCLM3
Huh7
c
Tube formation
Control
Co-culture+DMSO
Co-culture+GW9662
Co-culture+T0070907
HUVEC

Supplementary Figure 8 | PPARγ effects HCC progression through regulating *MMP9*⁺ TAMs differentiation assessed by PPARγ inhibition in THP-1 cell culture.

a Migration and **b** invasion abilities of HCCLM3 and Huh7 cells treated with THP-1 macrophages cultured alone (Control), cocultured with HCC cells (Co-culture+DMSO), or cocultured in the presence of the PPARγ inhibitors GW9662 (Co-culture+GW9662) and T0070907 (Co-culture+T0070907). **c** Tubes formation of human umbilical vein endothelial cells (HUVECs) treated with different sets of THP-1 macrophages: Control, Co-culture+DMSO, Co-culture+GW9662 and Co-culture+T0070907. In **a**–**c**, the experiment was repeated independently at least 3 times with similar results. The scale bars represent 20 μm. Source data are provided as a Source Data file.

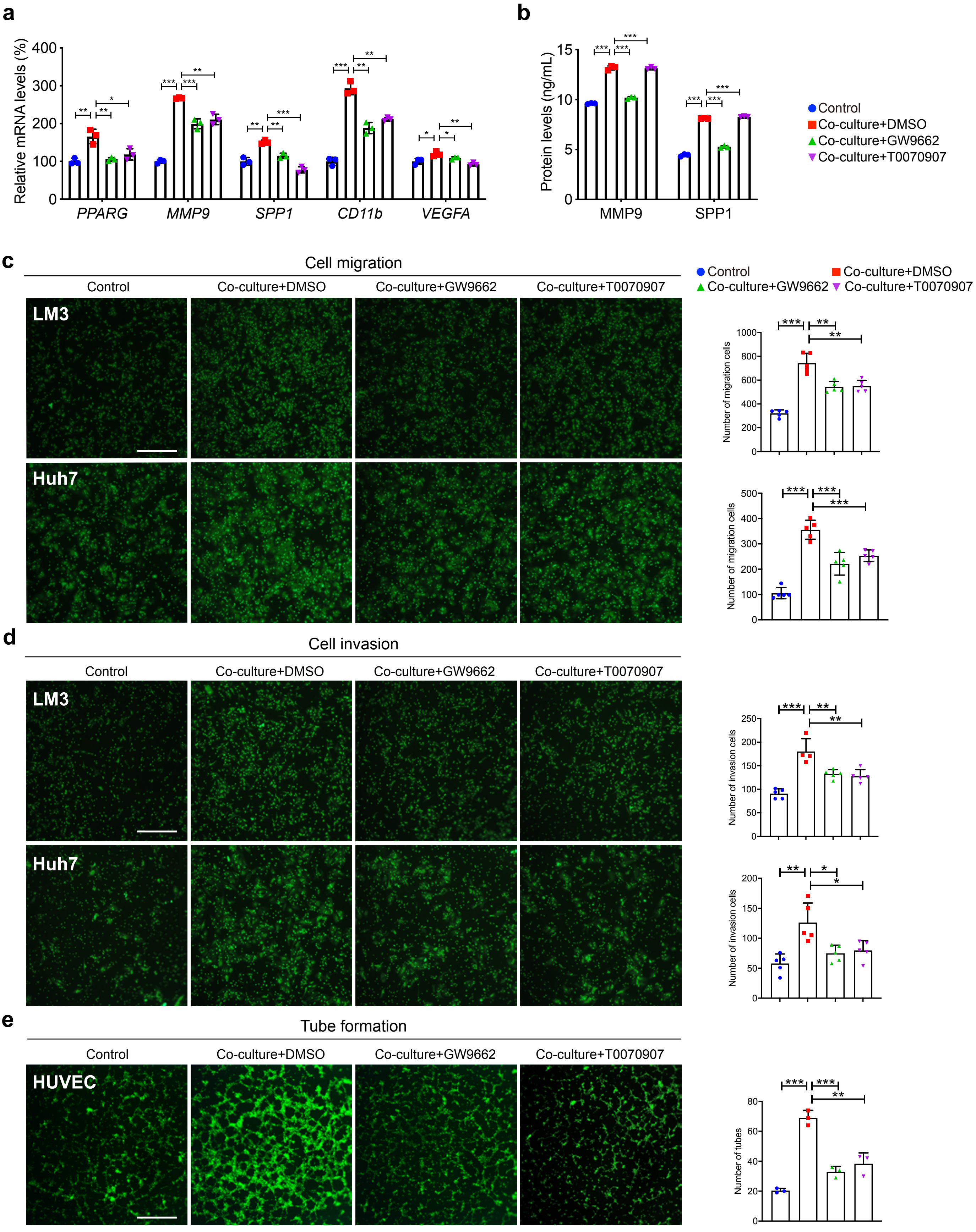

a
Relative mRNA levels (%)
400
300
200
100
0
PPARG
MMP9
SPP1
CD11b
VEGFA
b
Protein levels (ng/mL)
15
10
5
0
MMP9
SPP1
Control
Co-culture+DMSO
Co-culture+GW9662
Co-culture+T0070907
c
Cell migration
Control
Co-culture+DMSO
Co-culture+GW9662
Co-culture+T0070907
LM3
Huh7
Number of migration cells
d
Cell invasion
Control
Co-culture+DMSO
Co-culture+GW9662
Co-culture+T0070907
LM3
Huh7
Number of invasion cells
e
Tube formation
Control
Co-culture+DMSO
Co-culture+GW9662
Co-culture+T0070907
HUVEC
Number of tubes

Supplementary Figure 9 | PPARγ effects HCC progression through regulating *MMP9*$^{+}$ TAMs differentiation assessed by PPARγ inhibition in primary *MMP9*$^{+}$ TAMs culture.

a Expression levels of *MMP9*$^{+}$ TAMs markers measured by quantitative reverse transcription real-time polymerase chain reaction (qRT-PCR) in primary *MMP9*$^{+}$ TAMs cultured alone (Control), co-cultured with HCCLM3 cells (Co-culture+DMSO), or co-cultured in the presence of the PPARγ inhibitors GW9662 (Co-culture+GW9662) or T0070907 (Co-culture+T0070907) (n = 3 biological replicates). **b** Protein levels of *MMP9*$^{+}$ TAMs markers MMP9 and SPP1 measured by enzyme-linked immunosorbent assay (ELISA) in the culture media of primary *MMP9*$^{+}$ TAMs of different groups (n = 3 biological replicates). **c** Migration and **d** invasion abilities of HCCLM3 and Huh7 cells treated with THP-1 macrophages cultured alone (Control), cocultured with HCC cells (Co-culture+DMSO), or cocultured in the presence of the PPARγ inhibitors GW9662 (Co-culture+GW9662) and T0070907 (Co-culture+T0070907) (n = 5 biological replicates). **e** Tubes formation of human umbilical vein endothelial cells (HUVECs) treated with different sets of THP-1 macrophages: Control, Co-culture+DMSO, Co-culture+GW9662 and Co-culture+T0070907 (n = 3 biological replicates). The scale bars represent 20 μm. In **a**–**e**, error bars indicate mean ± sd and the statistical significances were determined by Student's *t* test. $^{*}P < 0.05$, $^{**}P < 0.01$, $^{***}P < 0.001$ and $^{****}P < 0.0001$. Source data are provided as a Source Data file.

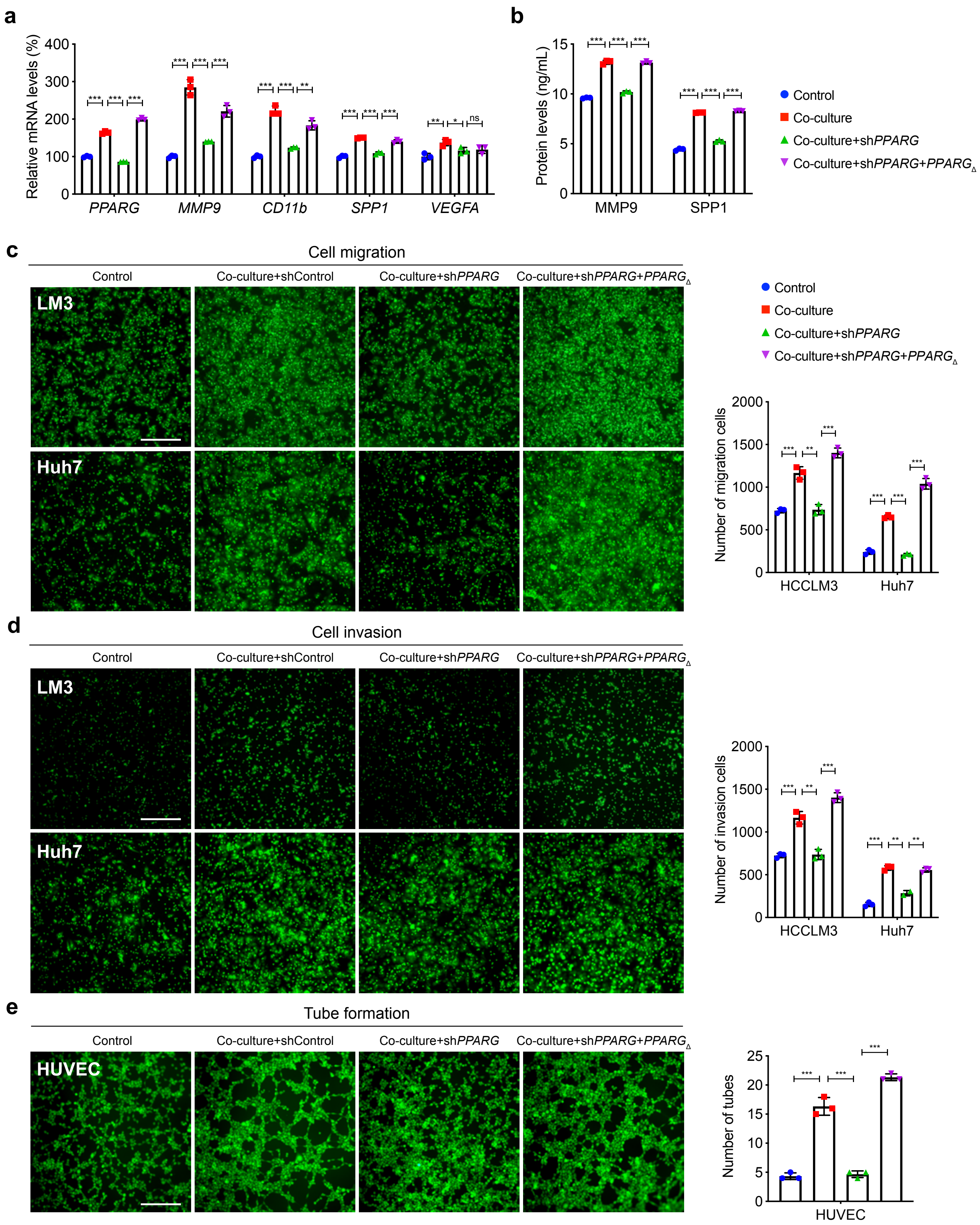

a
Relative mRNA levels (%)
PPARG
MMP9
CD11b
SPP1
VEGFA
b
Protein levels (ng/mL)
MMP9
SPP1
Control
Co-culture
Co-culture+shPPARG
Co-culture+shPPARG+PPARGΔ
c
Cell migration
Control
Co-culture+shControl
Co-culture+shPPARG
Co-culture+shPPARG+PPARGΔ
LM3
Huh7
Number of migration cells
HCCLM3
Huh7
d
Cell invasion
Number of invasion cells
e
Tube formation
HUVEC
Number of tubes

Supplementary Figure 10 | PPARγ effects HCC progression through regulating *MMP9*⁺ TAMs differentiation assessed by *PPARG* knockdown in THP-1 cell culture.

a Expression levels of *MMP9*$^+$ TAMs markers measured by quantitative reverse transcription real-time polymerase chain reaction (qRT-PCR) in THP-1 macrophages cultured alone (Control), cocultured with HCC cells (Coculture), or *PPARG* knockdown before the coculture (Coculture+sh*PPARG*) or rescue of *PPARG* expression in *PPARG* knockdown THP-1 macrophages before the coculture (Coculture+sh*PPARG*+sh$PPARG_{SM}$) (n = 3 biological replicates). **b** Protein levels of *MMP9*$^+$ TAMs markers MMP9 and SPP1 measured by enzyme-linked immunosorbent assay (ELISA) in the culture media of primary *MMP9*$^+$ TAMs of different groups (n = 3 biological replicates). **c** Migration and **d** invasion abilities of LM3 and Huh7 cells treated with THP-1 macrophages cultured alone (Control), cocultured with HCC cells (Coculture), or *PPARG* knockdown before the coculture (Coculture+sh*PPARG*) or rescue of *PPARG* expression in *PPARG* knockdown THP-1 macrophages before the coculture (Coculture+sh*PPARG*+sh$PPARG_{SM}$) (n = 3 biological replicates). **e** Number of tubes formed by human umbilical vein endothelial cells (HUVECs) treated with different sets of THP-1 macrophages: Control, Coculture, Coculture+sh*PPARG* and Coculture+sh*PPARG*+sh$PPARG_{SM}$ (n = 3 biological replicates). The scale bars represent 20 μm. In **a**–**e**, error bars indicate mean ± sd and the statistical significances were determined by two-sided Student's *t* test. $^{*}P < 0.05$, $^{**}P < 0.01$, $^{***}P < 0.001$ and $^{****}P < 0.0001$. Source data are provided as a

Source Data file.

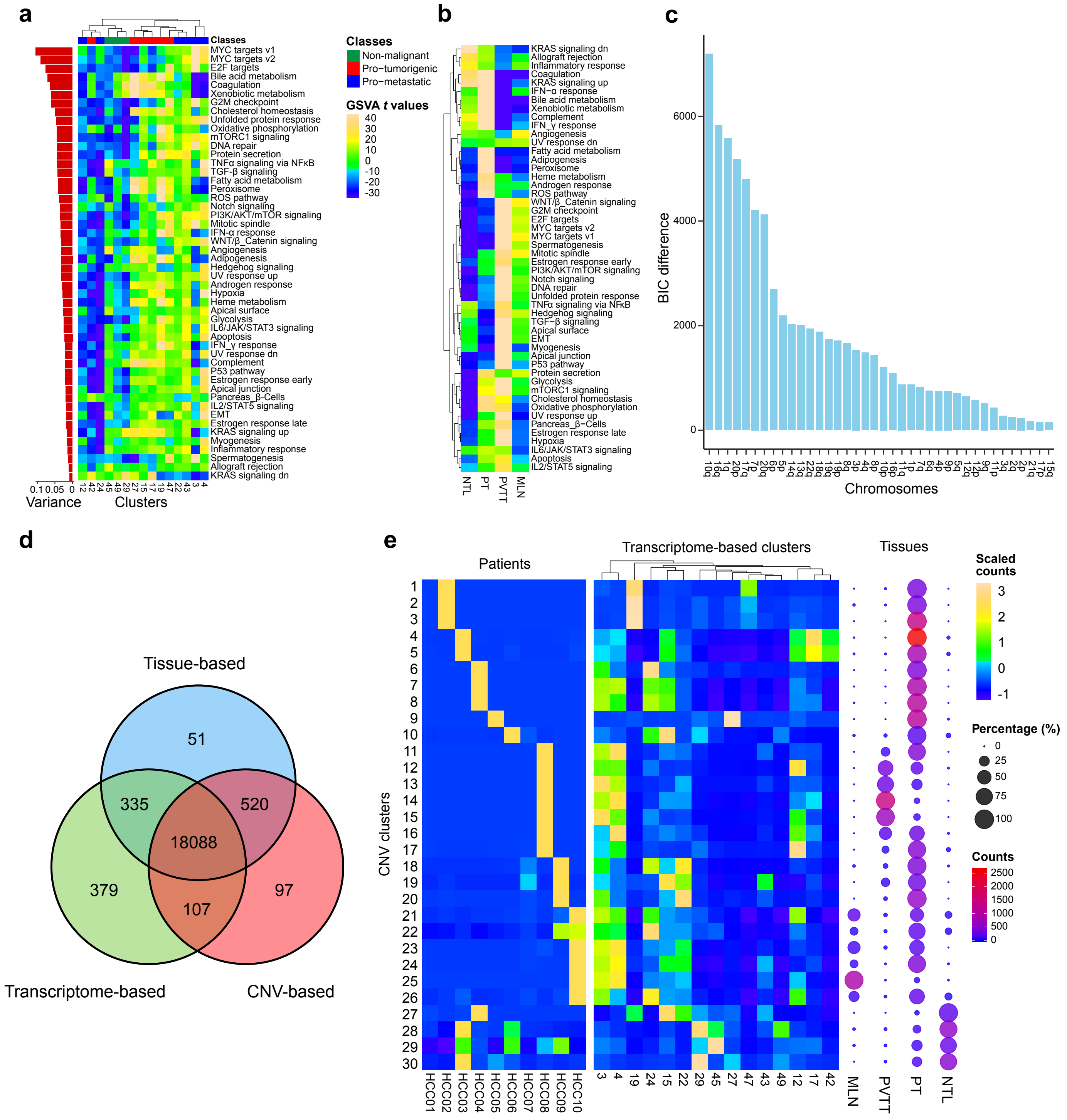
a
Classes
MYC targets v1
MYC targets v2
E2F targets
Bile acid metabolism
Coagulation
Xenobiotic metabolism
G2M checkpoint
Cholesterol homeostasis
Unfolded protein response
Oxidative phosphorylation
mTORC1 signaling
DNA repair
Protein secretion
TNFα signaling via NFκB
TGF-β signaling
Fatty acid metabolism
Peroxisome
ROS pathway
Notch signaling
PI3K/AKT/mTOR signaling
Mitotic spindle
IFN-α response
WNT/β_Catenin signaling
Angiogenesis
Adipogenesis
Hedgehog signaling
UV response up
Androgen response
Hypoxia
Heme metabolism
Apical surface
Glycolysis
IL6/JAK/STAT3 signaling
Apoptosis
IFN_γ response
UV response dn
Complement
P53 pathway
Estrogen response early
Apical junction
Pancreas_β-Cells
IL2/STAT5 signaling
EMT
Estrogen response late
KRAS signaling up
Myogenesis
Inflammatory response
Spermatogenesis
Allograft rejection
KRAS signaling dn
0.1 0.05 0
Variance
Clusters
Classes
Non-malignant
Pro−tumorigenic
Pro−metastatic
GSVA t values
40
30
20
10
0
-10
-20
-30
b
KRAS signaling dn
Allograft rejection
Inflammatory response
Coagulation
KRAS signaling up
IFN−α response
Bile acid metabolism
Xenobiotic metabolism
Complement
IFN_γ response
Angiogenesis
UV response dn
Fatty acid metabolism
Adipogenesis
Peroxisome
Heme metabolism
Androgen response
ROS pathway
WNT/β_Catenin signaling
G2M checkpoint
E2F targets
MYC targets v2
MYC targets v1
Spermatogenesis
Mitotic spindle
Estrogen response early
PI3K/AKT/mTOR signaling
Notch signaling
DNA repair
Unfolded protein response
TNFα signaling via NFκB
Hedgehog signaling
TGF−β signaling
Apical surface
EMT
Myogenesis
Apical junction
P53 pathway
Protein secretion
Glycolysis
mTORC1 signaling
Cholesterol homeostasis
Oxidative phosphorylation
UV response up
Pancreas_β-Cells
Estrogen response late
Hypoxia
IL6/JAK/STAT3 signaling
Apoptosis
IL2/STAT5 signaling
NTL
PT
PVTT
MLN
c
BIC difference
6000
4000
2000
0
Chromosomes
d
Tissue-based
51
335
520
18088
379
107
97
Transcriptome-based
CNV-based
e
Patients
Transcriptome-based clusters
Tissues
CNV clusters
HCC01
HCC02
HCC03
HCC04
HCC05
HCC06
HCC07
HCC08
HCC09
HCC10
MLN
PVTT
PT
NTL
Scaled counts
3
2
1
0
-1
Percentage (%)
0
25
50
75
100
Counts
2500
2000
1500
1000
500
0

Supplementary Figure 11 | Intratumoral heterogeneity of the malignant hepatocytes.

Differences of pathway activities among **a** different hepatocyte/cholangiocyte clusters and **b** hepatocytes at different tissue types. Shown are *t* values from a linear model. Pathways are sorted according to the variance of activities across all hepatocytes. The non-malignant, pro-tumorigenic and the pro-metastatic hepatocyte clusters are indicated by colors at the top of the heatmap. GSVA, gene set variation analysis; dn, down; EMT, epithelial-mesenchymal transition; ROS, reactive oxygen species; UV, ultraviolet; v1, version 1; v2, version 2. **c** Barplot of the difference of Bayesian Information Criterion (BIC) between a one-component and a two-component Gaussian Mixture Model. **d** Venn plot showing the overlap of malignant cells identified based on sample tissue types, gene expression profiles and single-cell copy number variation (CNV) profiles. **e** Distribution of cells in the 30 CNV-based clusters identified by unsupervised *k*-means clustering based on the CNV profiles across patients (left), the transcriptome-based clusters (center) and tissue types (right). Among these 30 CNV clusters, 26 clusters exhibited significant CNVs that are relevant to HCC, while the other four clusters did not exhibit any significant CNV, indicating they were associated to malignant and non-malignant cells, respectively. Heatmaps (left and center) show scaled cell counts. Dot size indicates the percentage of cells from the respective tissue types, and dot color indicates cell counts. NTL, non-tumor liver; PT, primary tumor; PVTT, portal vein tumor thrombus; MLN, metastatic lymph node. Source data are provided as a Source Data file.

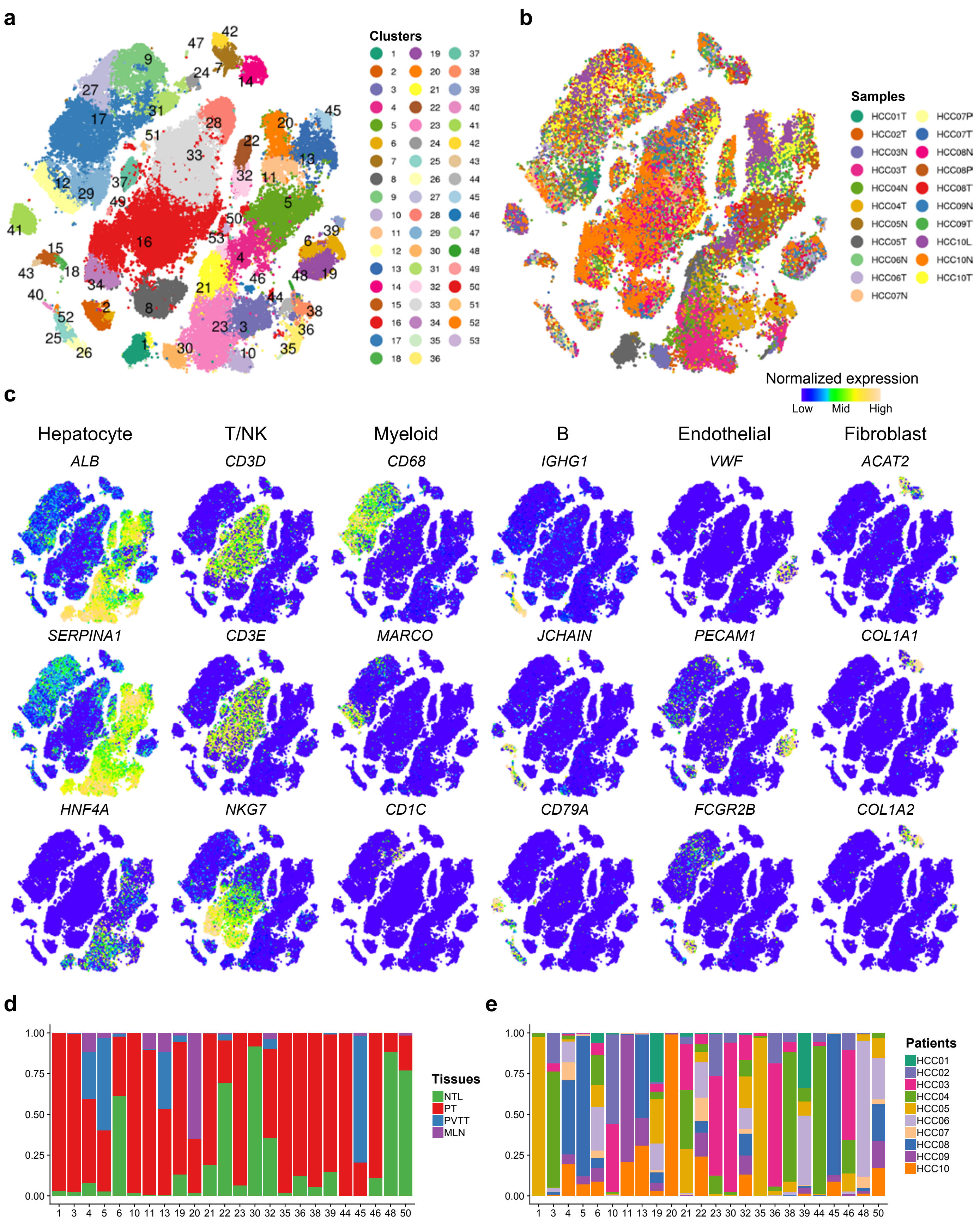
a
Clusters
b
Samples
HCC01T
HCC02T
HCC03N
HCC03T
HCC04N
HCC04T
HCC05N
HCC05T
HCC06N
HCC06T
HCC07N
HCC07P
HCC07T
HCC08N
HCC08P
HCC08T
HCC09N
HCC09T
HCC10L
HCC10N
HCC10T
c
Normalized expression
Low
Mid
High
Hepatocyte
T/NK
Myeloid
B
Endothelial
Fibroblast
ALB
CD3D
CD68
IGHG1
VWF
ACAT2
SERPINA1
CD3E
MARCO
JCHAIN
PECAM1
COL1A1
HNF4A
NKG7
CD1C
CD79A
FCGR2B
COL1A2
d
Tissues
NTL
PT
PVTT
MLN
e
Patients
HCC01
HCC02
HCC03
HCC04
HCC05
HCC06
HCC07
HCC08
HCC09
HCC10

Supplementary Figure 12 | Batch correction and cell clustering by the mutual nearest neighbor algorithm.

The T-distributed Stochastic Neighbor Embedding (tSNE) plot of all cells with batch correction by the mutual nearest neighbor (MNN) algorithm implemented by the Scran package, colored by **a** cell clusters and **b** samples, respectively. **c** The tSNE plots showing the expression levels of signature genes of six major cell types, colored by gene expression. **d** The fractions of cells in hepatocyte clusters from different tissue types. NTL, non-tumor liver; PT, primary tumor; PVTT, portal vein tumor thrombus; MLN, metastatic lymph node. **e** The fractions of cells from different patients across hepatocyte clusters. Source data are provided as a Source Data file.

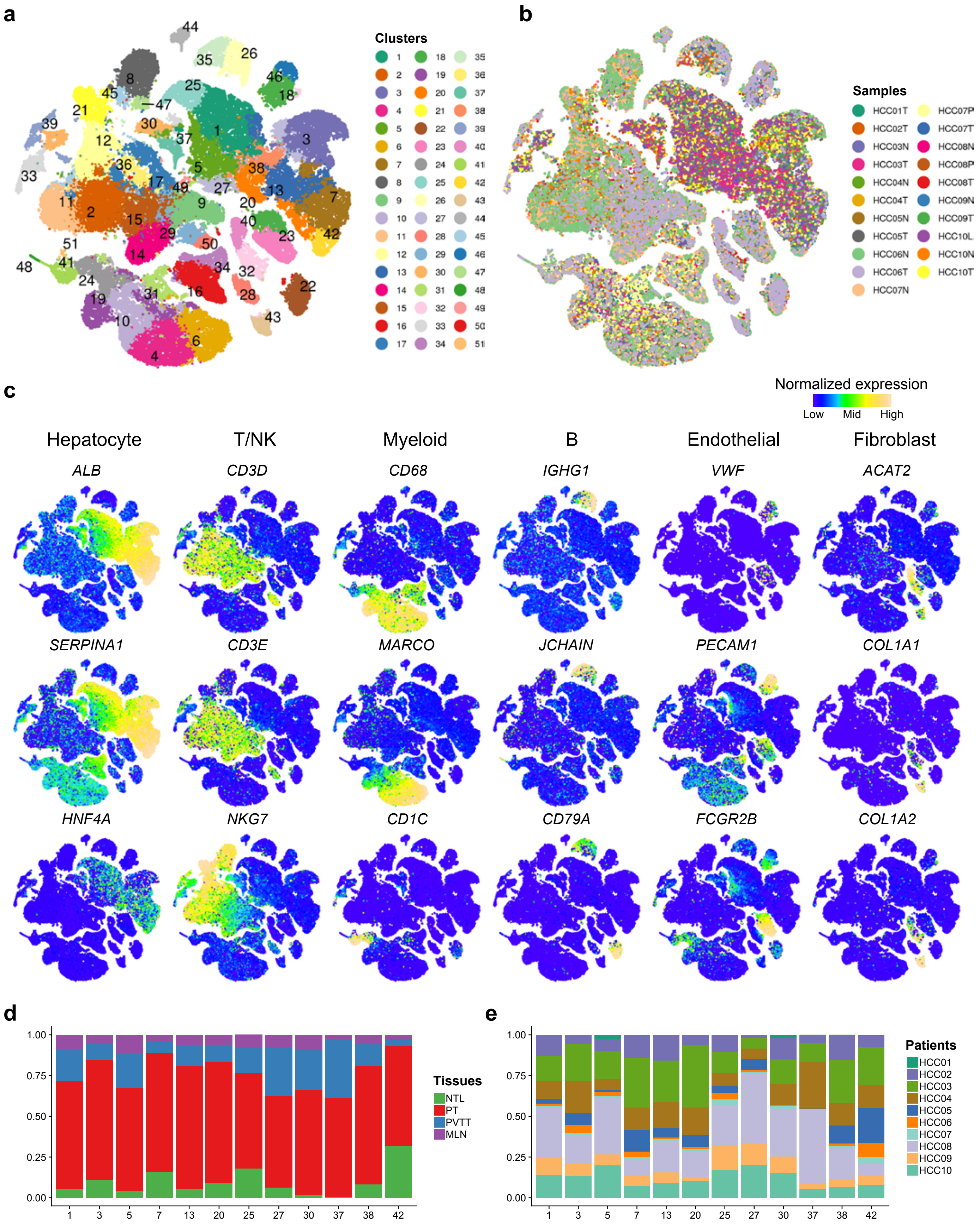
a
b
Clusters
Samples
HCC01T
HCC02T
HCC03N
HCC03T
HCC04N
HCC04T
HCC05N
HCC05T
HCC06N
HCC06T
HCC07N
HCC07P
HCC07T
HCC08N
HCC08P
HCC08T
HCC09N
HCC09T
HCC10L
HCC10N
HCC10T
c
Normalized expression
Low
Mid
High
Hepatocyte
T/NK
Myeloid
B
Endothelial
Fibroblast
ALB
CD3D
CD68
IGHG1
VWF
ACAT2
SERPINA1
CD3E
MARCO
JCHAIN
PECAM1
COL1A1
HNF4A
NKG7
CD1C
CD79A
FCGR2B
COL1A2
d
Tissues
NTL
PT
PVTT
MLN
e
Patients
HCC01
HCC02
HCC03
HCC04
HCC05
HCC06
HCC07
HCC08
HCC09
HCC10

Supplementary Figure 13 | Batch correction and cell clustering by the anchor-based algorithm.

The T-distributed Stochastic Neighbor Embedding (tSNE) plot of all cells with batch correction by the anchor-based algorithm implemented by the Seurat v3 package, colored by **a** cell clusters and **b** samples, respectively. **c** The tSNE plots showing the expression levels of signature genes of six major cell types, colored by gene expression. **d** The fractions of cells in hepatocyte clusters from different tissue types. NTL, non-tumor liver; PT, primary tumor; PVTT, portal vein tumor thrombus; MLN, metastatic lymph node. **e** The fractions of cells from different patients across hepatocyte clusters. Source data are provided as a Source Data file.

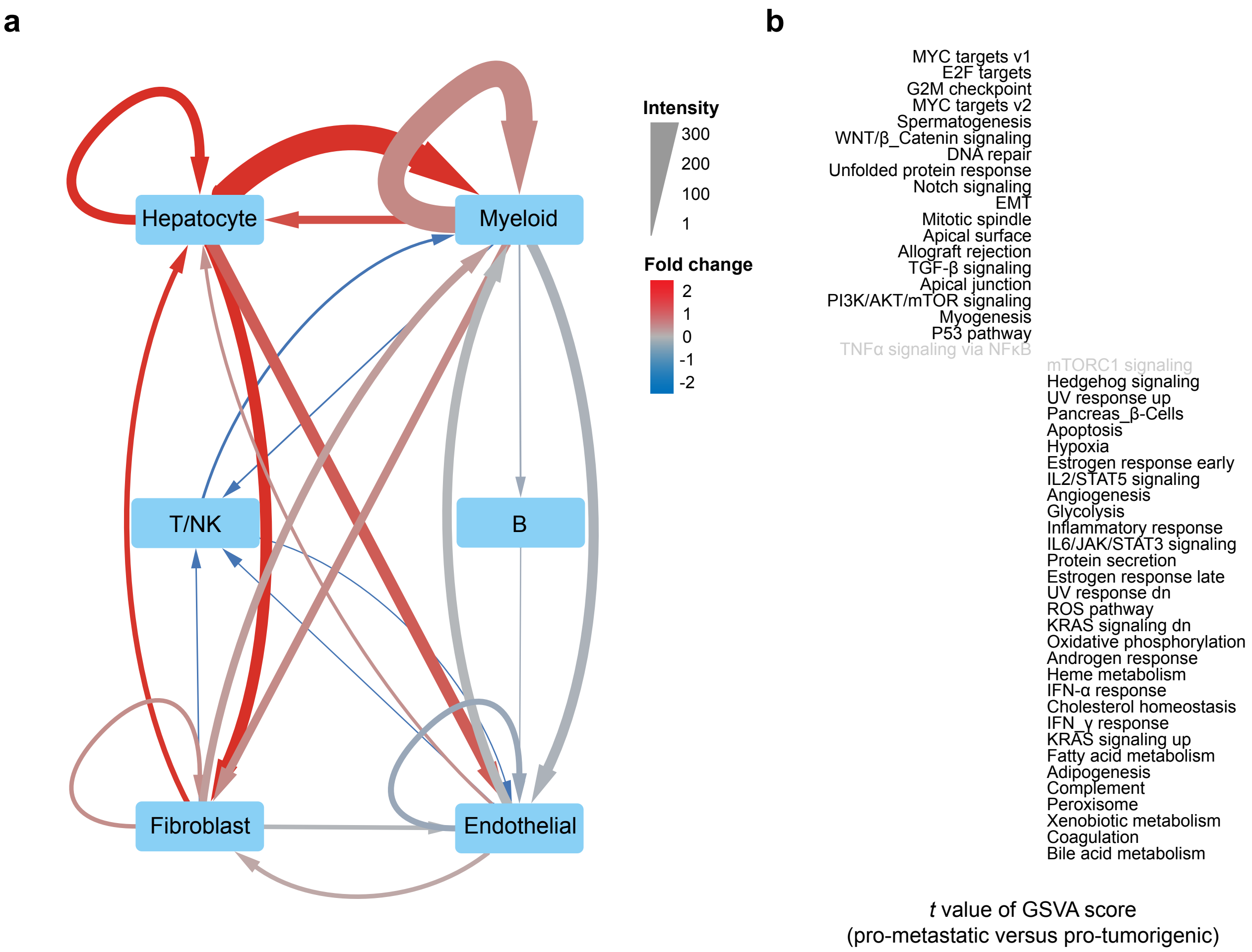
a
Hepatocyte
Myeloid
T/NK
B
Fibroblast
Endothelial
Intensity
300
200
100
1
Fold change
2
1
0
-1
-2
b
MYC targets v1
E2F targets
G2M checkpoint
MYC targets v2
Spermatogenesis
WNT/β_Catenin signaling
DNA repair
Unfolded protein response
Notch signaling
EMT
Mitotic spindle
Apical surface
Allograft rejection
TGF-β signaling
Apical junction
PI3K/AKT/mTOR signaling
Myogenesis
P53 pathway
TNFα signaling via NFκB
mTORC1 signaling
Hedgehog signaling
UV response up
Pancreas_β-Cells
Apoptosis
Hypoxia
Estrogen response early
IL2/STAT5 signaling
Angiogenesis
Glycolysis
Inflammatory response
IL6/JAK/STAT3 signaling
Protein secretion
Estrogen response late
UV response dn
ROS pathway
KRAS signaling dn
Oxidative phosphorylation
Androgen response
Heme metabolism
IFN-α response
Cholesterol homeostasis
IFN_γ response
KRAS signaling up
Fatty acid metabolism
Adipogenesis
Complement
Peroxisome
Xenobiotic metabolism
Coagulation
Bile acid metabolism
t value of GSVA score
(pro-metastatic versus pro-tumorigenic)

Supplementary Figure 14 | The enriched ligand-receptor interactions among the cell types in the microenvironment of PVTT tissues.

a A directed network showing the differential ligand-receptor (L-R) interaction intensities among the six major cell types (hepatocytes, T/NK, myeloid, B, endothelial cells and fibroblasts) between the portal vein tumor thrombus (PVTT) and non-tumor liver tissues. The averaged L-R interaction intensities in PVTT and non-tumor liver tissues are represented by edge width. Fold change of L-R interaction intensities is represented by edge color, with red denoting up-regulated and blue denoting down-regulated in PVTT as compared to non-tumor liver tissues. **b** Differences in pathway activities scored per cell by GSVA between pro-metastatic (n = 11,624) and pro-tumorigenic hepatocytes (n = 7,285). Shown are *t* values from a linear model. Significantly up- and down-regulated pathways (adjusted *P* value < 0.01) were marked with red and blue bars, respectively, and grey bars denote non-significant pathways. dn, down; EMT, epithelial-mesenchymal transition; ROS, reactive oxygen species; UV, ultraviolet; v1, version 1; v2, version 2. Source data are provided as a Source Data file.

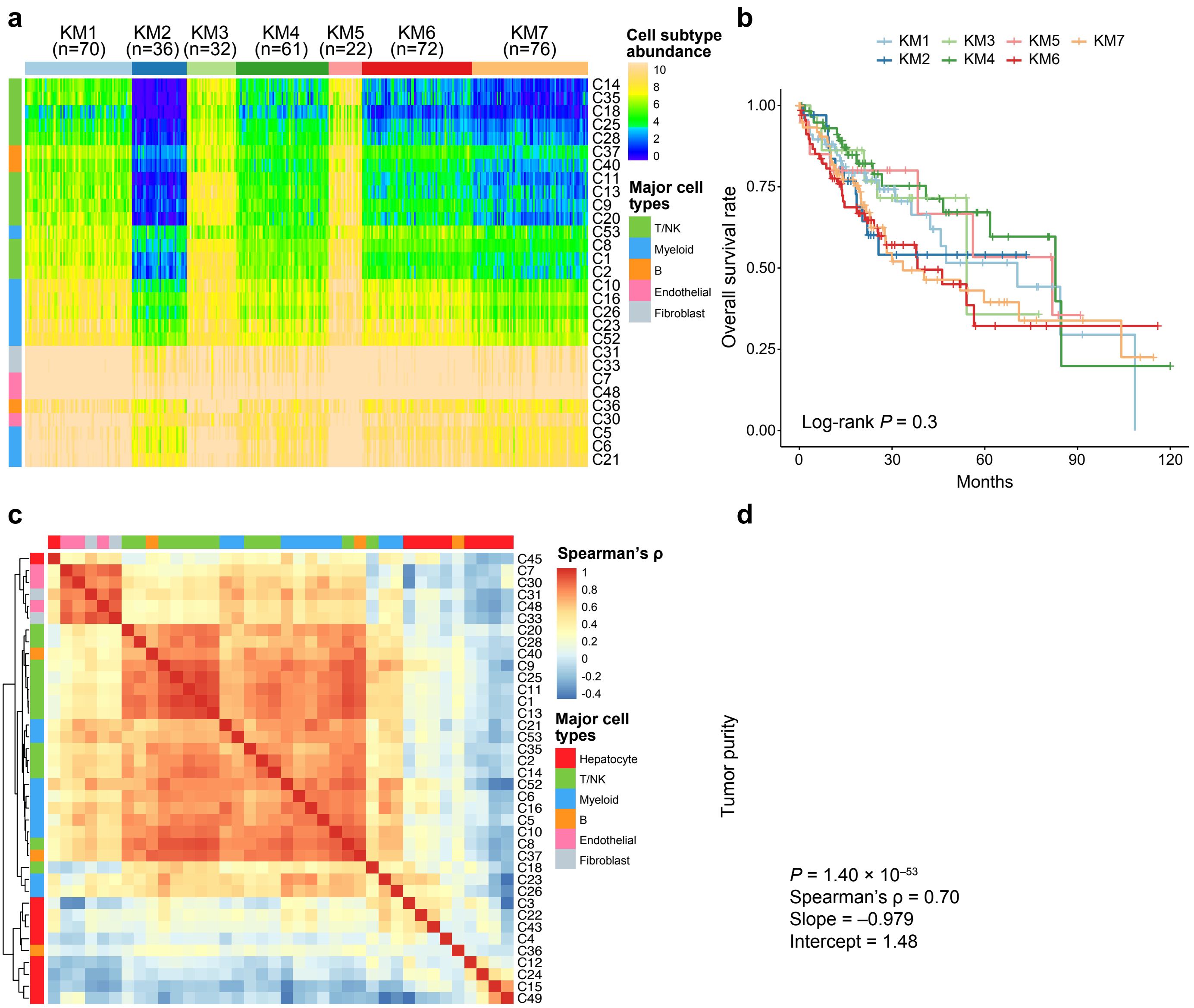

a
KM1 (n=70)
KM2 (n=36)
KM3 (n=32)
KM4 (n=61)
KM5 (n=22)
KM6 (n=72)
KM7 (n=76)
Cell subtype abundance
10
8
6
4
2
0
Major cell types
T/NK
Myeloid
B
Endothelial
Fibroblast
C14 C35 C18 C25 C28 C37 C40 C11 C13 C9 C20 C53 C8 C1 C2 C10 C16 C26 C23 C52 C31 C33 C7 C48 C36 C30 C5 C6 C21
b
KM1 KM2 KM3 KM4 KM5 KM6 KM7
Overall survival rate
1.00
0.75
0.50
0.25
0.00
Log-rank P = 0.3
0
30
60
90
120
Months
c
Spearman's ρ
1
0.8
0.6
0.4
0.2
0
-0.2
-0.4
Major cell types
Hepatocyte
T/NK
Myeloid
B
Endothelial
Fibroblast
C45 C7 C30 C31 C48 C33 C20 C28 C40 C9 C25 C11 C1 C13 C21 C53 C35 C2 C14 C52 C6 C16 C5 C10 C8 C37 C18 C23 C26 C3 C22 C43 C4 C36 C12 C24 C15 C49
d
Tumor purity
P = 1.40 × 10^{-53}
Spearman's ρ = 0.70
Slope = –0.979
Intercept = 1.48

Supplementary Figure 15 | Identification of the TME subtypes of HCCs using cell type deconvolution analyses.

a Heatmap showing seven TME classes (KM1-7) of the HCCs identified by *k*-means clustering using the estimated abundance of non-malignant cell subtypes. Rows represent the 29 patient-shared non-malignant cell subtypes identified by the scRNA-seq data. Columns represent the 369 HCC patients in the TCGA-LIHC cohort. **b** Kaplan-Meier survival analyses showing that the seven TME classes without normalization exhibit mixed survival outcomes. The statistical significance was determined by log-rank test. **c** The correlation between the estimated abundance of 40 patient-shared malignant and non-malignant cell subtypes. Two highly correlated blocks are related to immune cells (T/NK, myeloid and B cells) and stromal cells (endothelial cells and fibroblasts) respectively. Shown are the Spearman's ρ between the estimated abundance of cell types across 369 HCC patients. Major cell types of these clusters are indicated by colors at the left and top of the heatmap. **d** Scatter plot showing the correlation between the standardized intensity of non-malignant cells and tumor purity across 369 HCC tumors from the TCGA-LIHC cohort. The solid line is the result of fitting a linear regression model. The statistical significance was determined by one-way analysis of variance (ANOVA). Source data are provided as a Source Data file.
