# Supplementary material for: A single-cell atlas of the multicellular ecosystem of primary and metastatic hepatocellular carcinoma
Source: Nat Commun. 2022 Aug 6;13:4594. doi: 10.1038/s41467-022-32283-3 (PMC9357016; doi:10.1038/s41467-022-32283-3)
Supplement: Supplementary file 2 — Description of Additional Supplementary Files [file 41467_2022_32283_MOESM2_ESM.docx]

**Description of Additional Supplementary Files**

Supplementary Data 1

Description: Clinical characteristics of enrolled HCC patients.

File Name: Supplementary Data 2

Description: Statistics of single-cell RNA sequencing data.

File Name: Supplementary Data 3

Description: Cell count across patients and tissue types and top 10 mark genes of 53 cell clusters.

File Name: Supplementary Data 4

Description: Significantly up- and down-regulated genes in exhausted CTLs (KM5) compared to other CTLs (KM1–4).

File Name: Supplementary Data 5

Description: Significantly up- and down-regulated genes in pre-exhausted CTLs (KM4) compared to non-exhausted CTLs (KM1–3).

File Name: Supplementary Data 6

Description: Primers used for RT-qPCR of PPARG and MMP9+ TAMs signature genes.

File Name: Supplementary Data 7

Description: Signature genes up-regulated in pro-metastatic hepatocytes as compared to pro-tumorigenic hepatocytes.

File Name: Supplementary Data 8

Description: Significant L-R interactions in non-tumor liver tissues.

File Name: Supplementary Data 9

Description: Significant L-R interactions in primary tumor tissues.

File Name: Supplementary Data 10

Description: Significant L-R interactions in PVTT tissues.

File Name: Supplementary Data 11

Description: TME subtypes and related classification information of 369 HCC patients in the TCGA-LIHC cohort.
