# Supplementary material for: A single-cell atlas of the multicellular ecosystem of primary and metastatic hepatocellular carcinoma
Source: Nat Commun. 2022 Aug 6;13:4594. doi: 10.1038/s41467-022-32283-3 (PMC9357016; doi:10.1038/s41467-022-32283-3)
Supplement: Supplementary file 4 — Reporting Summary [file 41467_2022_32283_MOESM4_ESM.pdf]

nature portfolio

Corresponding author(s): Gangqiao Zhou

Last updated by author(s): Jun 4, 2022

Reporting Summary

Nature Portfolio wishes to improve the reproducibility of the work that we publish. This form provides structure for consistency and transparency in reporting. For further information on Nature Portfolio policies, see our Editorial Policies and the Editorial Policy Checklist.

Statistics

For all statistical analyses, confirm that the following items are present in the figure legend, table legend, main text, or Methods section.

n/a	Confirmed	
☐	☒	The exact sample size (*n*) for each experimental group/condition, given as a discrete number and unit of measurement
☐	☒	A statement on whether measurements were taken from distinct samples or whether the same sample was measured repeatedly
☐	☒	The statistical test(s) used AND whether they are one- or two-sided<br>*Only common tests should be described solely by name; describe more complex techniques in the Methods section.*
☒	☐	A description of all covariates tested
☐	☒	A description of any assumptions or corrections, such as tests of normality and adjustment for multiple comparisons
☐	☒	A full description of the statistical parameters including central tendency (e.g. means) or other basic estimates (e.g. regression coefficient) AND variation (e.g. standard deviation) or associated estimates of uncertainty (e.g. confidence intervals)
☐	☒	For null hypothesis testing, the test statistic (e.g. *F*, *t*, *r*) with confidence intervals, effect sizes, degrees of freedom and *P* value noted<br>*Give P values as exact values whenever suitable.*
☒	☐	For Bayesian analysis, information on the choice of priors and Markov chain Monte Carlo settings
☒	☐	For hierarchical and complex designs, identification of the appropriate level for tests and full reporting of outcomes
☐	☒	Estimates of effect sizes (e.g. Cohen's *d*, Pearson's *r*), indicating how they were calculated

Our web collection on statistics for biologists contains articles on many of the points above.

Software and code

Policy information about availability of computer code

Data collection	No software was used for the collection of the data in the paper.
Data analysis	Cellranger (v2.2)<br>Seurat (v2)<br>Seurat (v3)<br>Scran (v1.14.6)<br>destiny (v3.0.1)<br>RaceID (v0.1)<br>Velocyto.py<br>Velocyto.R<br>GSVA (v1.34)<br>GSEA (v7)<br>pySCENIC(0.10.4)<br>CONICS (0.1)<br>NMI (v2)<br>limma (v3.42.2)<br>InForm (v2.2.1)<br>BD FACSDIVA (v6)<br>GRNboost2 (v1)

For manuscripts utilizing custom algorithms or software that are central to the research but not yet described in published literature, software must be made available to editors and reviewers. We strongly encourage code deposition in a community repository (e.g. GitHub). See the Nature Portfolio guidelines for submitting code & software for further information.

Data

Policy information about availability of data

All manuscripts must include a data availability statement. This statement should provide the following information, where applicable:

- Accession codes, unique identifiers, or web links for publicly available datasets
- A description of any restrictions on data availability
- For clinical datasets or third party data, please ensure that the statement adheres to our policy

The raw data of single-cell RNA sequencing generated in this study have been deposited in the European Genome-phenome Archive (EGA) database under accession code EGAC00001001616. Because all data submitted to the EGA must be subject to controlled access as defined by the original informed consents, researchers have to propose a scientific hypothesis to require access by contacting Prof. Gangqiao Zhou. The processed gene expression data of single-cell RNA sequencing is available at the Gene Expression Omnibus (GEO) database under accession code GSE149614. The raw and processed gene expression data of bulk RNA sequencing is available at the GEO database under accession code GSE168922. The L-R data used in this study are available in the Fantom5 and CellPhoneDB databases via https://fantom.gsc.riken.jp/5/suppl/Ramilowski_et_al_2015/ and https://www.cellphonedb.org/downloads. Analyzing, visualizing, and downloading of the single-cell transcriptome data is available at http://omic.tech/scrna-hcc/.

Field-specific reporting

Please select the one below that is the best fit for your research. If you are not sure, read the appropriate sections before making your selection.

☒ Life sciences ☐ Behavioural & social sciences ☐ Ecological, evolutionary & environmental sciences

For a reference copy of the document with all sections, see nature.com/documents/nr-reporting-summary-flat.pdf

Life sciences study design

All studies must disclose on these points even when the disclosure is negative.

Sample size	Ten patients with primary and/or metastatic HCC were enrolled and their non-tumor liver (NTL), primary tumor (PT), portal vein tumor thrombus (PVTT) and metastatic lymph node (MLN) tissues were collected for single-cell RNA sequencing (scRNA-seq) and/or multi-color immunohistochemistry (IHC) assay. Besides, sixteen additional HCC patients were enrolled and their NTL, PT, PVTT and MLN tissues were used for flow cytometry analysis and/or multi-color IHC assay.
Data exclusions	No data were excluded from the analysis.
Replication	For FACS and IHC assays, each experiment was repeated at least five times. For functional assays, each experiment was repeated at least three times. All these experiments were successful and data were used in the analysis.
Randomization	This is not relevant to this study for the HCC patients were not divided into groups during recruitment. Biological samples were randomly allocated into different groups during experiments.
Blinding	HCC patients were not divided into groups. Investigators were blinded to group allocation during data collection and/or analysis for experiments other than those involving patients.

Reporting for specific materials, systems and methods

We require information from authors about some types of materials, experimental systems and methods used in many studies. Here, indicate whether each material, system or method listed is relevant to your study. If you are not sure if a list item applies to your research, read the appropriate section before selecting a response.

Materials & experimental systems

n/a	Involved in the study	
☐	☒	Antibodies
☐	☒	Eukaryotic cell lines
☒	☐	Palaeontology and archaeology
☒	☐	Animals and other organisms
☐	☒	Human research participants
☒	☐	Clinical data
☒	☐	Dual use research of concern

Methods

n/a	Involved in the study	
☒	☐	ChIP-seq
☐	☒	Flow cytometry
☒	☐	MRI-based neuroimaging

Antibodies

Antibodies used	7-AAD (FACS) Thermo Fisher Cat# 559925

Anti-CD45 (FACS) BD Biosciences Cat# 560178
Anti-CD68 (FACS) BD Biosciences Cat# 564943
Anti-CD11b (FACS) BD Biosciences Cat# 557701
Anti-MMP9 (FACS) CST Cat# 27647
Anti-TREM2 (FACS) R&D Systems, Cat# FAB17291A
Anti-CD3 (FACS) BD Biosciences Cat# 555342
Anti-CD8 (FACS) BD Biosciences Cat# 564526
Anti-PD-1 (FACS) BD Biosciences Cat# 564323
Anti-CD45RA antibody (IHC) ZSBIO Cat# ZM0053
Anti-CD45RO antibody (IHC) ZSBIO Cat# ZM0055
Anti-CD4 antibody (IHC) Abcam Cat# ab133616
Anti-CD8 antibody (IHC) Abcam Cat# ab17147
Anti-CCR7 antibody (IHC) Abcam Cat# ab1657
Anti-CD20 antibody (IHC) Abcam Cat# ab9475
Anti-ALB antibody (IHC) Proteintech Cat# 16475-1-AP
Anti-MIF antibody (IHC) Abcam Cat# ab55445
Anti-CXCL10 antibody (IHC) Proteintech Cat# 10937-1-AP
Anti-CD68 antibody (IHC) ZSGB-BIO Cat# ZM-0060
Anti-MM9 antibody (IHC) Proteintech Cat# 10375-2-AP
Anti-CD74 antibody (IHC) Abcam Cat# ab9514
Anti-CCR7 antibody (IHC) Proteintech Cat# 55425-1-AP
Anti-CXCR3 antibody (IHC) Proteintech Cat# 60065-1-Ig

Validation

All antibodies were validated by the manufacturers for antigen specificity and species reactivity as shown in the data sheets and attached references for each catalogue number. Antibodies were additionally validated for specificity and sensitivity and titrated in pre-experiments by using positive and negative control samples. In detail:
7-AAD (FACS) Thermo Fisher Cat# 559925
>https://www.thermofisher.cn/order/catalog/product/00-6993-50?adobe_mc=MCMID%7C82674438189264299853548014011351050572%7CMCAID%3D307679B7CB6928CF-400016440C77FF5D%7CMCORGID%3D5B135A0C5370E6B40A490D44%40AdobeOrg%7CTS=1614293705
Anti-CD45 (FACS) BD Biosciences Cat# 560178
>https://www.bdbiosciences.com/en-us/products/reagents/flow-cytometry-reagents/research-reagents/single-color-antibodies-ruo/apc-h7-mouse-anti-human-cd45.560274
Anti-CD68 (FACS) BD Biosciences Cat# 564943
>https://www.bdbiosciences.com/content/dam/bdb/products/global/reagents/flow-cytometry-reagents/research-reagents/single-color-antibodies-ruo/564943_base/pdf/564943.pdf
Anti-CD11b (FACS) BD Biosciences Cat# 557701
>https://www.bdbiosciences.com/en-us/products/reagents/flow-cytometry-reagents/research-reagents/single-color-antibodies-ruo/bv711-mouse-anti-human-cd11b.742641
Anti-MMP9 (FACS) CST Cat# 27647
>https://www.cellsignal.com/products/antibody-conjugates/mmp-9-d6o3h-xp-rabbit-mab-pe-conjugate/27647
Anti-TREM2 (FACS) R&D Systems, Cat# FAB17291A
>https://www.rndsystems.com/cn/products/human-mouse-trem2-apc-conjugated-antibody-237920_fab17291a
Anti-CD3 (FACS) BD Biosciences Cat# 555342
>https://www.bdbiosciences.com/en-nz/products/reagents/flow-cytometry-reagents/research-reagents/single-color-antibodies-ruo/apc-mouse-anti-human-cd3.555342
Anti-CD8 (FACS) BD Biosciences Cat# 564526
>https://www.bdbiosciences.com/en-us/products/reagents/flow-cytometry-reagents/research-reagents/single-color-antibodies-ruo/bb515-mouse-anti-human-cd8.564526
Anti-PD-1 (FACS) BD Biosciences Cat# 564323
>https://www.bdbiosciences.com/en-nz/products/reagents/flow-cytometry-reagents/research-reagents/single-color-antibodies-ruo/bv421-mouse-anti-human-cd279-pd-1.564323
Anti-CD45RA antibody (IHC) ZSBIO Cat# ZM0053
>http://www.zsbio.com/product/ZM-0053
Anti-CD45RO antibody (IHC) ZSBIO Cat# ZM0055
>http://www.zsbio.com/product/ZM-0055
Anti-CD4 antibody (IHC) Abcam Cat# ab133616
>https://www.abcam.com/cd4-antibody-epr6855-ab133616.html
Anti-CD8 antibody (IHC) Abcam Cat# ab17147
>https://www.abcam.com/cd8-alpha-antibody-c8144b-ab17147.html
Anti-CCR7 antibody (IHC) Abcam Cat# ab1657
>https://www.abcam.com/ccr7-antibody-ab1657.html
Anti-CD20 antibody (IHC) Abcam Cat# ab9475
>https://www.abcam.com/cd20-antibody-l26-ab9475.html
Anti-ALB antibody (IHC) Proteintech Cat# 16475-1-AP
>https://www.ptglab.com/products/ALB-Antibody-16475-1-AP.htm
Anti-MIF antibody (IHC) Abcam Cat# ab55445
>https://www.abcam.com/mif-antibody-2a10-4d3-ab55445.html
Anti-CXCL10 antibody (IHC) Proteintech Cat# 10937-1-AP

>https://www.ptglab.com/products/CXCL10-Antibody-10937-1-AP.htm
Anti-CD68 antibody (IHC) ZSGB-BIO Cat# ZM-0060
>http://www.zsbio.com/product/ZM-0060
Anti-MM9 antibody (IHC) Proteintech Cat# 10375-2-AP
>https://www.ptglab.com/products/MMP9-Antibody-10375-2-AP.htm
Anti-CD74 antibody (IHC) Abcam Cat# ab9514
>https://www.abcam.com/cd74-antibody-ln2-ab9514.html
Anti-CCR7 antibody (IHC) Proteintech Cat# 55425-1-AP
>https://www.ptglab.com/products/CCR7-Antibody-55425-1-AP.htm
Anti-CXCR3 antibody (IHC) Proteintech Cat# 60065-1-Ig
>https://www.ptglab.com/products/CXCR3B-Antibody-60065-1-Ig.htm

Eukaryotic cell lines

Policy information about cell lines

Cell line source(s)	THP-1 cell line was obtained from China Center for Type Culture Collection (GDC100)<br>HCCLM3 cell line was obtained from China Center for Type Culture Collection (GDC0289)<br>Huh7 cell line was obtained from China Center for Type Culture Collection (GDC0134)<br>HUVEC cell line was obtained from China Center for Type Culture Collection (GDC166)
Authentication	All cell lines were authenticated by short tandem repeat (STR) analysis at China Center for Type Culture Collection.
Mycoplasma contamination	All cell lines were tested negative for Mycoplasma contamination.
Commonly misidentified lines<br>(See ICLAC register)	No commonly misidentified cell lines were used in the study.

Human research participants

Policy information about studies involving human research participants

Population characteristics	Briefly, all the HCC patients were newly diagnosed, pathologically confirmed, and proved not to have other types of cancer. The diagnosis of HCC was made by either positive histologic findings or an elevated serum α-fetoprotein (AFP) level (≥ 400 ng/mL) combined with at least one positive image on angiography, sonography, and/or high-resolution contrast computed tomography. Among the 26 HCC patients, 16 were chronic HBV carriers, who were positive for both hepatitis B surface antigen (HBsAg) and antibody immunoglobulin G to hepatitis B core antigen (HBcAb) for at least 6 months, and 2 were chronic HCV carriers, who were positive for hepatitis C antibody (HCV-Ab). Additionally, all the subjects included in this study were negative for antibodies to hepatitis D virus or human immunodeficiency virus; and had no other types of liver disease, including autoimmune hepatitis, toxic hepatitis, and primary biliary cirrhosis or Budd-Chiari syndrome. None of the patients was treated with chemotherapy, radiotherapy or any other anti-tumor medicines prior to tumor resection.
Recruitment	Ten patients with primary and/or metastatic HCC were enrolled between July 2018 and December 2018 at the Chinese PLA General Hospital (Beijing, China), and their non-tumor liver (NTL), primary tumor (PT), portal vein tumor thrombus (PVTT) and metastatic lymph node (MLN) tissues were collected for single-cell RNA sequencing (scRNA-seq) and/or multi-color immunohistochemistry (IHC) assay. Besides, sixteen additional HCC patients were enrolled between September 2019 and April 2022 from the same hospital, and their NTL, PT, PVTT and MLN tissues were used for flow cytometry analysis and/or multi-color IHC assay.
Ethics oversight	This study was approved by the Research and Ethical Committee of Chinese PLA General Hospital (Beijing, China) and Beijing Institute of Radiation Medicine (Beijing, China).

Note that full information on the approval of the study protocol must also be provided in the manuscript.

Flow Cytometry

Plots

Confirm that:

- [x] The axis labels state the marker and fluorochrome used (e.g. CD4-FITC).
- [x] The axis scales are clearly visible. Include numbers along axes only for bottom left plot of group (a 'group' is an analysis of identical markers).
- [x] All plots are contour plots with outliers or pseudocolor plots.
- [x] A numerical value for number of cells or percentage (with statistics) is provided.

Methodology

Sample preparation	Following surgical resection, the fresh tissues of PT, NTL, PVTT and MLN were immediately transferred to pre-cooled MACS Tissue Storage Solution (Miltenyi Biotech, Germany) and were shipped at 4°C. For each sample, ~1 g tissue was used for dissociation, and the remaining tissue, if any, was fixed in formalin for 48 hours (h) and embedded in paraffin for subsequent

	immunohistochemistry analysis. For dissociation, the tissue was minced using the surgical scalpels and further disintegrated using the Liver Dissociation Kit (Miltenyi Biotech, Germany) and the gentleMACS Dissociator (Miltenyi Biotech, Germany) according to manufacturer's instructions. The resulting single-cell suspension was filtered sequentially through sterile 70-µm and 40-µm cell strainers. The cell suspension was stained for viability with 25 mM cisplatin (Enzo Life Sciences, USA) in a 1-minute (min) pulse before quenching with 10% FBS. The single-cell suspensions were then used for subsequent droplet-based scRNA-seq or flow cytometry analysis.
Instrument	gentleMACS Dissociator (Miltenyi Biotech, Germany)<br>Aria II flow cytometer (BD Biosciences, USA)
Software	BD FACSDIVA software (BD Biosciences, USA)<br>FlowJo software (FlowJo, USA)<br>HALO pathology software (Indica Labs, USA)
Cell population abundance	We analyzed 1,000,000-2,000,000 cells in total per sample. Post-sort analysis are also plotted in the supplement figures.
Gating strategy	For sorting macrophages, the expression levels of CD45, CD68, CD11b, MMP9 and TREM2 were gated by their negative controls of unstained cells and positive controls of cells stained by each antibody. For sorting the MMP9+ TAMs from the PT and NTL tissues, samples were gated for CD45+CD68+CD11b+MMP9+. For sorting the TREM2+ TAMs from the PT and NTL tissues, samples were gated for CD45+CD68+TREM2+. Besides, the other macrophages except for MMP9+ TAMs (referred to as non-MMP9+ TAMs) in the tumor tissues and the whole macrophage populations in the NTL tissues were sorted as negative controls by gating for CD45+CD68+CD11b–/CD45+CD68+MMP9– and CD45+CD68+ respectively.<br>For sorting T cells, The expression levels of CD3, CD8 and PD1 were gated by their negative controls of unstained cells and positive controls of cells stained by each antibody. Cells were analyzed using the BD FACSDIVA software (BD Biosciences, USA) and FlowJo software (FlowJo, USA).

☒ Tick this box to confirm that a figure exemplifying the gating strategy is provided in the Supplementary Information.
